# Genome-Wide Identification of *Osmanthus fragrans* Histone Modification Genes and Analysis of Their Expression during the Flowering Process and under Azacytidine and Ethylene Treatments

**DOI:** 10.3390/plants13060777

**Published:** 2024-03-09

**Authors:** Hui Xia, Yingting Zhang, Xiang Chen, Xiangling Zeng, Xuan Cai, Zeqing Li, Hongguo Chen, Jie Yang, Jingjing Zou

**Affiliations:** 1 National Forestry and Grassland Administration Engineering Research Center for Osmanthus Fragrans, Hubei University of Science and Technology, Xianning 437100, China; xiahui@njfu.edu.cn (H.X.); ytzhang0308@163.com (Y.Z.); cengxiangling@hbust.edu.cn (X.Z.); caixuan@hbust.edu.cn (X.C.); lizeqing@hbust.edu.cn (Z.L.); chen_hongguo1969@163.com (H.C.); 2 Osmanthus Innovation Center of National Engineering Research Center for Floriculture, Hubei University of Science and Technology, Xianning 437100, China; 3 Hubei Key Laboratory of Agricultural Bioinformatics, College of Informatics, Huazhong Agricultural University, Wuhan 430070, China; cx652@webmail.hzau.edu.cn; 4 Research Center for Osmanthus Fragrans, Xianning Research Academy of Industrial Technology of Osmanthus Fragrans, Xianning 437100, China

**Keywords:** *Osmanthus fragrans*, histone modifications, *HM* gene family, expression analysis, flowering process, azacytidine treatment, ethylene treatment

## Abstract

Histone acetylation and methylation, governed by various histone modification (HM) gene families, are vital for plant biological processes. However, there are limited studies that have explored *HMs* in ornamental horticultural trees, including sweet osmanthus (*Osmanthus fragrans*). We performed genome-wide search and identified 208 *OfHMs*, encompassing 81 histone methyltransferases (OfHMTs), 51 histone demethylases (OfHDMs), 49 histone acetyltransferases (OfHATs) and 27 histone deacetylases (HDACs). Our comprehensive analysis covered chromosome locations, gene structures, conserved domains, *cis*-acting elements, phylogenetic comparisons, protein interaction networks and functional enrichment pathways for these gene families. Additionally, tandem and fragment replications were unveiled as contributors to the expansion of *OfHMs*, with some genes exhibiting positive selection. Furthermore, we examined *OfHM* expression profiles across various tissues and flowering stages, and under 5′-azacytidine (Aza) and ethylene treatments. Most *OfHMs* displayed heightened expression in leaves, and were downregulated during the flower opening and senescence stages, including *OfPRMTs*, *OfHDTs*, *OfHDAs*, *OfSRTs*, *OfJMJs* and *OfHAGs*; 75.86% and 80.77% of the differentially expressed *OfHMs* were upregulated after Aza and ethylene treatments, including *OfHAGs*, *OfHDAs* and *OfSDGs*. This study offers a comprehensive analysis of the *OfHM* gene family, which indicated their potential involvement in ethylene and Aza responses, and in the flowering process. These findings provide valuable insights into the role of *OfHMs* in flowering and stress responses.

## 1. Introduction

Histone modifications (HMs) are essential for modulating gene expression through modifying chromatin structure and stability, thereby influencing diverse biological processes in plants [1,2,3]. These processes include not only biological growth and development, but also the response to stress. The primary types of HMs encompass histone acetylation, methylation, phosphorylation, ubiquitination, SUMOylation and ADP-ribosylation [4]. Among these types, histone acetylation and methylation have been extensively studied [5,6] and are recognized as two pivotal and widespread epigenetic regulatory mechanisms that significantly impact gene expression.

Histone acetylation, under the control of histone acetyltransferases (HATs) and histone deacetylases (HDACs), involves the modification of lysine residues in histone proteins through the addition or removal of acetyl groups [7,8]. HAT-induced acetylation promotes the opening of local chromatin, facilitating the binding of transcription factors and increasing gene expression, while HDAC-associated acetylation is often associated with gene repression [9,10,11]. Histone methylation, catalyzed by histone demethylases (HDMs) and histone methyltransferases (HMTs), involves the addition or removal of methyl groups from the histone tails [7]. Combinatorial binding modules in histone recognizers recognize this modification, ultimately influencing chromatin structure and gene expression [4]. The impact of histone methylation on the initiation and inhibition of gene expression is primarily determined by the position of the methylation. For instance, trimethylation of lysine at position 4 on histone H3 (H3K4me3) is linked to the initiation of gene expression, while trimethylation of lysine at position 27 on histone H3 (H3K27me3) is correlated with repressed gene expression [12,13].

Histone acetylation and methylation modifications are predominantly governed by various members of the *HM* gene family, a group that has been discovered and characterized in several plant species, such as tomato (*Solanum lycopersicum*) [14], apple (*Malus domestica*) [8], litchi (*Litchi chinensis*) [15], sweet orange (*Citrus sinensis*) [16], rice (*Oryza sativa*) [17] and strawberry (*Fragaria vesca*) [18]. The *HM* gene family encompasses the *HAT*, *HDAC*, *HMT* and *HDM* gene families [7,8], each of which contains distinct subfamilies. Specifically, the *HAT* family includes four subfamilies. *HAG* includes histone acetylases with a GCN5-, ELP3- and HAT1-like domain, *HAM* involves the MOZ-YBF2 (MYST) domain, *HAC* features the HAT_KAT11 domain, and *HAF* is associated with the TATA-binding protein-related factor TAF [7,8]. The *HDAC* family consists of three subfamilies: HD2 (*HDT*), RPD3/HDA1 (*HDA*) and silent information regulator 2 (*SRT*) [19]. The *HMT* family comprises two subfamilies: the SET domain group (*SDG*) and protein arginine methyltransferases (*PRMT*), and the *HDM* family, which also includes two subfamilies, the SWIRM and C-terminal domain (*HDMA*) protein family and the JmjC domain protein family (*JMJ*) [7,8].

The functions of *HM* genes have been delineated, revealing their involvement in various plant processes [20,21]. These processes encompass growth and development, and stress responses, including photomorphogenesis [22], embryo development [23], seed germination and dormancy [2], flowering processes [8], fruit development [15,16,18], stress and defensive responses [3,16,17,18] and signaling responses to hormones [17]. In particular, *HM* genes play a pivotal role in crucial physiological processes associated with plant flowering, including flower induction, petal senescence and regulation of the flowering period. For instance, members of the *HAT* family, such as *AtHAM1* and *AtHAM2*, influence flowering time through epigenetic modification of FLOWERING LOCUS C (*FLC*) and MADS AFFECTING FLOWERING 3/4 (*MAF3/4*) chromatin through H4K5 acetylation [24]. *AtHAC1* promotes the *Arabidopsis* flowering process through epigenetic modifications to *FLC* upstream factors [25]. Within the *HDAC* family, *AtHDA6* and *AtHDA9* regulate the flowering locus D/VE (*FLD/FVE*), osmotically responsive gene 1 (*HOS1*), nuclear localized protein 22 of the AT hook motif (*AHL22*) and AGAMOUS-LIKE 9 (*AGL9*), thus influencing the flowering time of *Arabidopsis* [26,27]. The AtHDA19 complex directly regulates gibberellin (GA) signaling, impacting the expression of *FLC* and FLOWERING LOCUS T (*FT*) through a pathway that inhibits flowering [28]. In the *HDM* family, *AtJMJ14*, *AtJMJ15* and *AtJMJ18* regulate the flower opening time of *Arabidopsis* by modulating the trimethylation level of histone H3K4 [29,30,31]; *BcJMJ30*, a gene that encodes a histone demethylase with a jmjC domain, is involved in the development of pollen and fertilization of rape (*Brassica campestris*) [32]. The trithorax group gene (*TrxG*) *AtSDG25* of the *HMT* family, which is involved in H3K4 and H3K36 methylation, delays flowering by activating *FLC* expression [33]. However, the roles of *HMs* in ornamental horticultural trees, including sweet osmanthus (*Osmanthus fragrans*), have received limited attention.

*O. fragrans*, a distinguished member of the Oleaceae family, stands as one of the most renowned and traditional flowers in China. Celebrated for its intense fragrance [34], it is widely embraced in horticulture and landscaping. Beyond its ornamental value, it has found mature applications in the realms of food, cosmetics and medicines [34,35,36]. However, despite its versatility, it faces the challenge of a brief flowering period, which typically only lasts 2 to 3 days, limiting its ornamental and economic value [37,38]. The role of HMs in regulating the flowering period in *O. fragrans* remains elusive. This study used bioinformatics to identify *HM* gene members in the *O. fragrans* genome, conducting analyses of the gene structure, chromosomal location, phylogenetic comparisons, conserved protein domains, protein–protein interaction networks and functional enrichment. Furthermore, transcriptome data were utilized to analyze gene expression profiles in different tissues (stems, leaves and roots), during the flower opening and senescence processes, and under various treatment agents. These findings provide valuable information on *HM* genes during the flower opening and senescence stages of *O. fragrans*, thereby contributing essential information for comprehending the intricacies of flowering and senescence processes and enriching biological theories. Moreover, considering the significant economic importance of *O. fragrans,* research on *OfHM* genes holds potential application value in improving its yield and quality. This study could contribute to expanding our comprehensive understanding of plant life activities and provide new theoretical and practical support for plant genetic breeding and biotechnology applications.

## 2. Results

### 2.1. Identification and Characterization Analysis of HMs in the O. fragrans Genome

A total of 208 *OfHM*s were identified in the *O. fragrans* genome, which were classified into 81 *OfHMTs* (consisting of 12 *OfPRMT*s and 69 *OfSDG*s), 51 *OfHDMs* (encompassing 17 *OfHDMA*s and 34 *OfJMJ*s), 49 *OfHATs* (comprising 39 *OfHAG*s, 2 *OfHAM*s, 7 *OfHAC*s and 1 *OfHAF*) and 27 *OfHDACs* (including 17 *OfHDA*s, 3 *OfSRT*s and 7 *OfHDT*s) (Table S1). The OfHMTs exhibited lengths ranging from 258 to 2419 amino acids (aa), with molecular weights (MWs) varying from 29.30 to 276.20 kDa, isoelectric points (pIs) within the range of 4.55 to 9.16, and aliphatic indices within the range of 62.62 to 99.85 (Table S1). Among them, 83.95% of the OfHMTs exhibited an instability index >40, which is indicative of a prevalence of unstable proteins in this gene family (Table S1). The OfHDMs had lengths ranging from 135 to 2136 aa, MWs from 14.79 to 233.73 kDa, pIs from 4.89 to 9.43, and aliphatic indices from 65.85 to 94.89, with 88.24% being classified as unstable proteins (Table S1). The OfHATs exhibited lengths ranging from 105 to 1869 aa, with MWs from 11.73 to 212.34 kDa, pIs from 4.46 to 10.41, and aliphatic indices >60% (Table S1). The majority (67.35%) of them were characterized as unstable proteins (Table S1). OfHDACs, with lengths of 98–659 aa, MWs of 11.47–73.41 kDa, pIs of 4.11–10.55, and aliphatic indices ≥48.32, included unstable proteins among the OfSRTs (71.43%), OfHDTs (71.43%), and OfHDAs (47.06%) (Table S1). It is noteworthy that, excluding OfPRMT11, the other OfHMs possessed a grand average of hydropathicity (GRAVY) < 0, signifying hydrophilic properties (Table S1). Furthermore, in addition to OfSDG27 and OfHDA10, the remaining OfHMs lacked protein transmembrane domains (Table S1).

Most OfHM proteins (63.94%) were predicted to be localized in the nucleus (Table S1), and 21.63% of the proteins were identified on the cell membrane, encompassing the endomembrane system, plasma membrane and organelle membrane (Table S1). Furthermore, 11.54% of these proteins were assumed to be located within the chloroplast (Table S1).

### 2.2. Phylogenetic Analysis of HMs between O. fragrans and Arabidopsis

**Figure 1:**
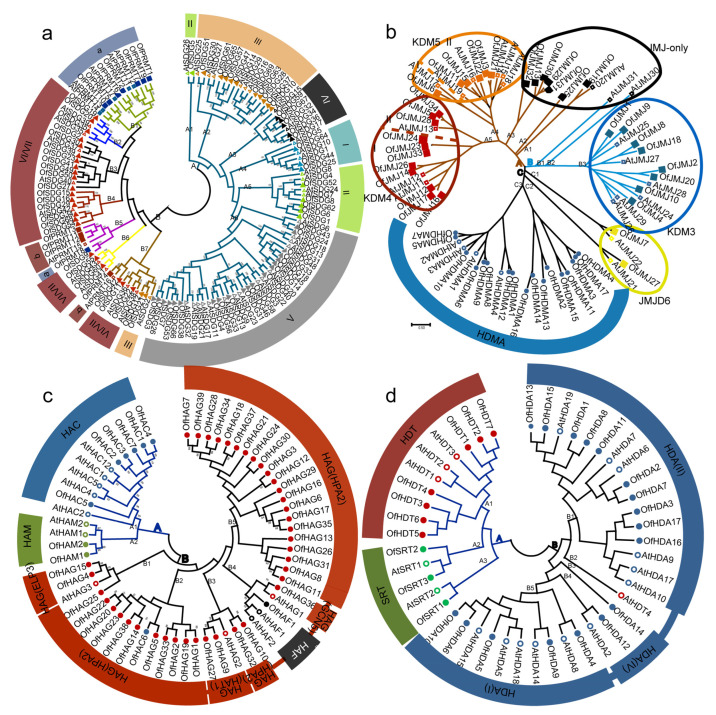
Phylogenetic analysis of histone modification *(HM)* genes between *O. fragrans* and *Arabidopsis*. (**a**) Histone methyltransferases (HMTs). Triangle symbols represent SET domain group (*SDG*) genes, and square symbols represent protein arginine methyltransferase (*PRMT*) genes. Solid symbols represent *O. fragrans* genes and hollow symbols represent *Arabidopsis* genes. I–VII represent the different types of *SDG* genes, and “a” and “b” represent different types of *PRMT* genes. (**b**) Histone demethylases (HDMs). Circle symbols represent SWIRM and C-terminal domain (*HDMA*) genes, and square symbols represent JmjC domain protein family (*JMJ*) genes. Solid symbols represent *O. fragrans* genes, and hollow symbols represent *Arabidopsis* genes. Different colored squares represent the various types of *JMJ* genes. (**c**) Histone demethylases (HDMAs) and (**d**) histone deacetylases (HDACs). Solid circles represent *O. fragrans* genes and hollow circles denote *Arabidopsis* genes. Different colors represent genes from different gene families.

To elucidate the phylogenetic relationships among HMs, we constructed four unrooted phylogenetic trees, specifically for HMTs, HDMs, HATs and HDACs, utilizing AtHMs and OfHMs. The classification of AtHMTs and OfHMTs revealed two distinct groups: A (A1–6) and B (B1–7), as illustrated in Figure 1a. Group A included types I (A4), II (A1, 5), IV (A3) and V (A6) SDGs, together with most type III (A2) SDGs, while Group B encompassed types VI/VII SDGs (B2–4, 6, 7), a few type III SDGs (B7) and types a (B1, 5) and b (B5, 7) PRMTs. The phylogenetic analysis of the AtHDMs and OfHDMs revealed a close clustering into three categories labeled A–C (Figure 1b). Specifically, branch C comprised all HDMAs (C3) and JMJD6-type genes (C1, 2), branch B included KDM3-type (B3) and some JMJ-only (B1, 2) genes, while branch A clustered KDM4-type (A5), KDM5-type (A4) and some JMJ-only (A1–3) genes. For the AtHATs and OfHATs, they were grouped into two branches: A and B, as shown in Figure 1c. Notably, with the exception of OfHAC6, the HACs (A1) and HAMs (A2) were placed on branch A, the HAFs were clustered in branch B4 and the HAGs were distributed throughout branch B. Similarly, all the HDACs were classified into two branches: A and B (Figure 1d). Specifically, excluding AtHDT4, branch A included the other HDTs (A1) and all SRTs (A2, 3), while branch B grouped the HDAs, with I- (B5), II- (B1) and IV-type HDAs (B3, 4).

### 2.3. Gene Structure and Conserved Motif Analyses of OfHMs

**Figure 2:**
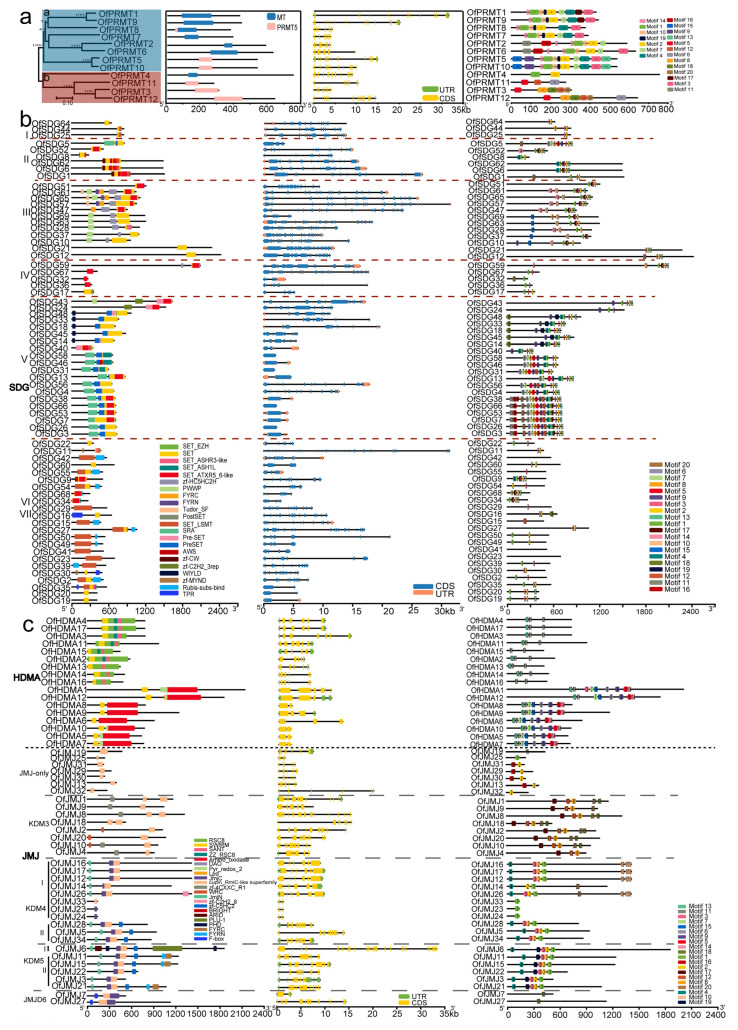
The domain, conserved structure and motif analyses of histone methylation modification genes in *O. fragrans*. (**a**) *OfPRMTs*; (**b**) *OfSDGs*. I–VII, represent different types of *OfSDG* genes. (**c**) *OfHDM*s. *OfHDM*s include *OfHDMAs* and *OfJMJs*.

Gene structure analyses provide valuable information on evolutionary relationships within gene families. Here, we explored the conserved domains, gene structures and motifs of 11 OfHM gene families, with the results presented in Figure 2. Among the OfPRMTs, which contained 7–20 coding sequences (CDSs), a consistent presence of uniform PRMT5/MT domains was observed (Figure 2a). The a-type OfPRMTs exhibited 7–11 different motifs, containing motifs 1, 2, 10 and 14; while the b-type OfPRMTs contained 2–6 motifs (Figure 2a). The OfSDGs, which contained 1 to 35 CDSs, featured SET domains and shared analogous motifs (Figure 2b). The I-type OfSDGs included motifs 2 and 3; the II-type OfSDGs, except for OfSDG5, contained the AWS domain and fewer motifs (3–6); the III-type OfSDGs, with the exceptions of OfSDG12, OfSDG21 and OfSDG51, embraced the zf-HC5HC2H and FYRC/PWWP domains, with the majority of genes possessing motifs 14, 15, 10, 1, 11, 3 and 2; most of the V-type OfSDGs also included the SRR/WIYLD and Pre-SET domains, with 3–15 motifs; most of the VI/VII-type OfSDGs had rubis-subs-bind/AWS/TPR domains, with 0–6 motifs (Figure 2b). The OfHDMAs, characterized by SWIRM domains, were categorized into two types based on the presence of RSC8 (Figure 2c). The I-type OfHDMAs contained 2 to 3 motifs, including motifs 11 and 13, while the II-type genes included 8–9 motifs, involving motifs 7, 15, 6, 9, 5 and 14 (Figure 2c). All the OfJMJs contained cupin_RmlC-like or JmjC domains (Figure 2c). The JMJ-only genes comprised 3–9 CDSs and 1–2 motifs; the KDM3-type genes included 2 cupin_RmlC-like domains or WRC and zf-4CXXC_R1 domains, with 8–20 CDSs and 3–5 motifs (such as 8, 12 and 17); the I-type KDM4 genes predominantly featured the JmjN domain, with 1–8 motifs; the II-type KDM4 contained JmjN and zf-C5HC2 domains, with 4 motifs (4, 3, 2 and 1); the KDM5-type genes included 5 motifs (motifs 4, 19, 3, 2 and 1) and the JmjN domain, with some genes containing zf-C5HC2, PLU-1, FYRN and FYRC domains; the JMJD6-type genes embraced the F-box domain and had only 1 motif (Figure 2c).

**Figure 3:**
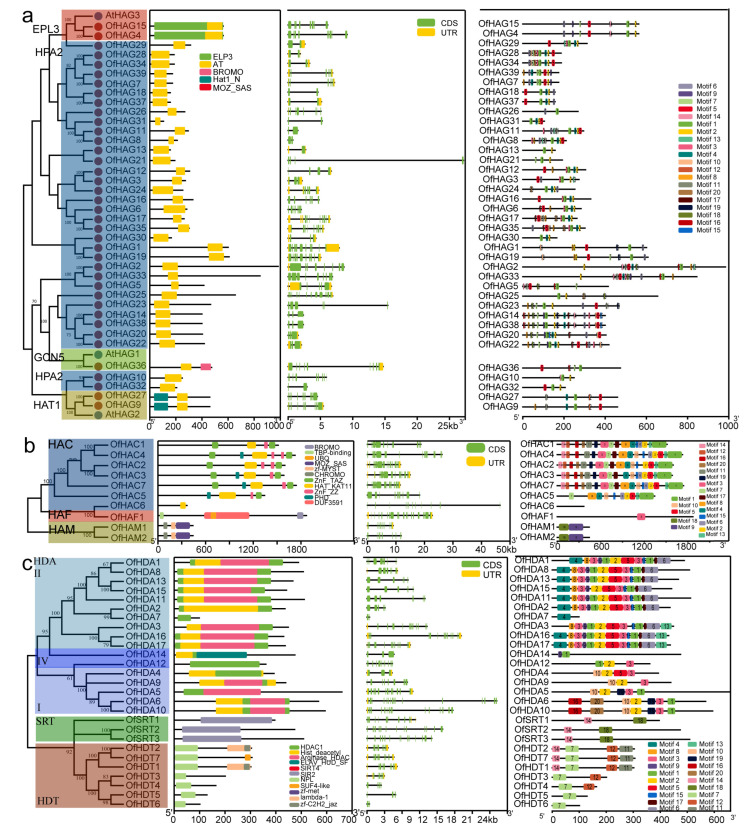
The analysis of domains, conserved structures and motifs of histone acetylation modification genes in *O. fragrans*. (**a**) *OfHAGs* (GCN5-, ELP3- and HAT1-like histone acetylases); (**b**) *OfHAMs* (MOZ-YBF2 (MYST) domain), *OfHACs* (HAT_KAT11 domain) and *OfHAFs* (TATA binding protein-related factor TAF); and (**c**) *OfHDMs*. II, IV, and I, represent different types of *OfHDM* genes.

The OfHAGs were distinguished by the presence of AT domains (Figure 3a). The EPL3-type HAGs (OfHAG4, 15) additionally featured the EPL3 domain, with eight CDSs and seven motifs, including motifs 1, 2, 5, 6, 7, 9 and 14 (Figure 3a). The GCN5-type HAG (OfHAG36) included a BROMO domain, with nine CDSs and four motifs, i.e., motifs 1, 5, 7 and 16 (Figure 3a). The Hat1_N-type HAGs (OfHAG9, 27) consisted of 10 CDSs and 5–6 motifs, such as 1, 4, 5, 11 and 12 (Figure 3a). The OfHACs featured the HAT_KAT11 domain, with 12 to 17 CDSs (Figure 3b). Except for OfHAC6, the other genes contained 10–18 motifs, including motifs 1, 13, 2, 6 and 7. OfHAF1 contained the DUF3591 domain, with 21 CDSs and motif 3 (Figure 3b). The OfHAMs contained CHROMO, zf-MYST and MOZ_SAS domains, with nine CDSs and motifs 9 and 18 (Figure 3b). The OfHDAs contained an HDAC1 domain, with 2–17 CDSs (Figure 3c). The I-type HDAs included 3–7 motifs, such as motifs 2, 3 and 10; the IV-type genes included 2–3 motifs; except for OfHDA7, which contained 1 motif, the other II-type HDAs included 12–13 motifs (Figure 3c). The OfHDTs contained an NPL domain, with 2–10 CDSs and motif 7 (Figure 3c). The OfSRTs, which featured an SIR2 domain, comprised 10–15 CDSs and motifs 14 and 18 (Figure 3c).

### 2.4. Cis-Acting Element Analysis of OfHMs

**Figure 4:**
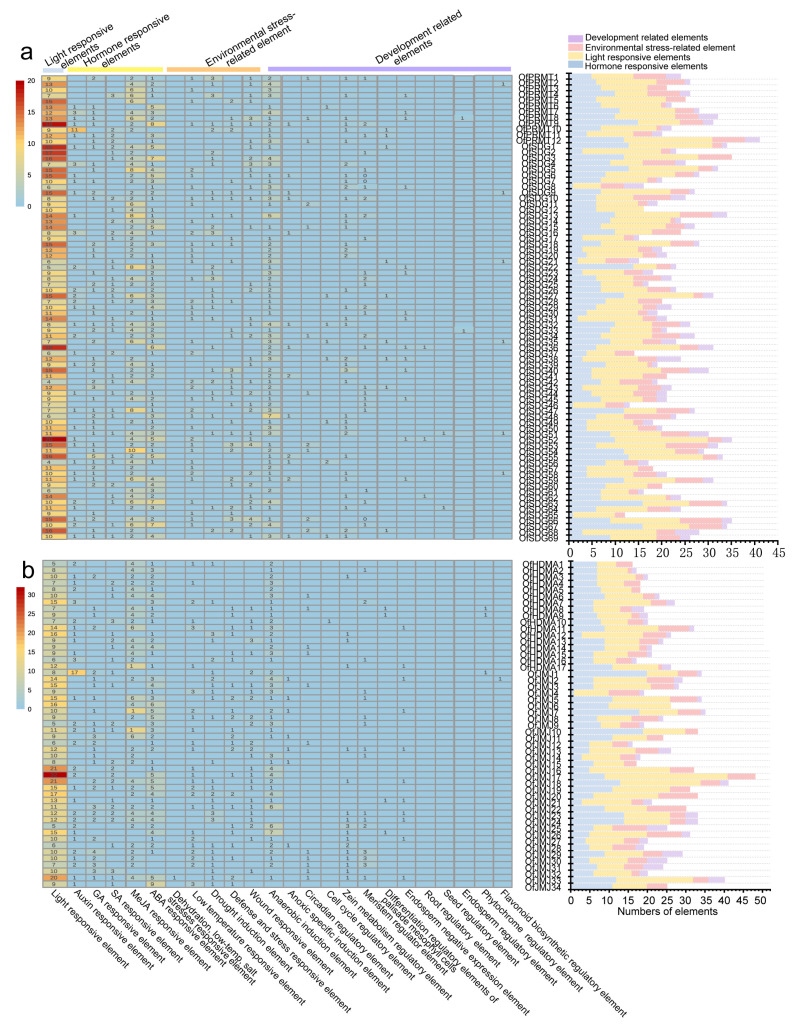
Analysis of *cis*-acting elements in the promoters of histone methylation modification genes. (**a**) *OfHMTs* and (**b**) *OfHDMs*. The figure on the left shows the different *cis*-acting elements, which are represented by boxes in different columns. The picture on the right provides the statistics regarding the number of each of the four kinds of *cis*-acting elements in the OfHM promoters.

**Figure 5:**
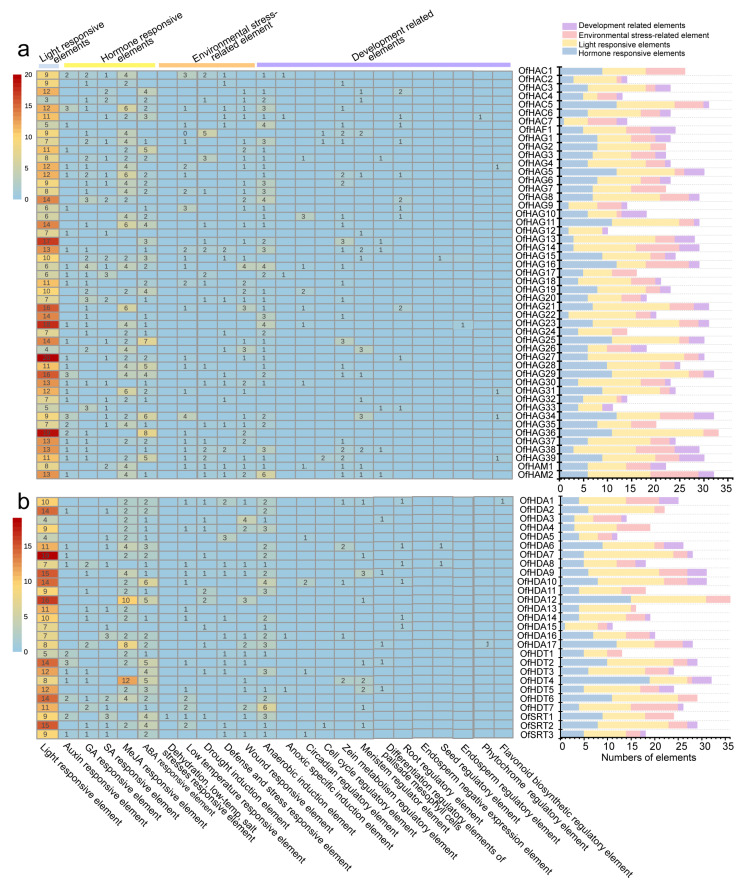
Analysis of *cis*-acting elements in the promoters of histone acetylation modification genes. (**a**) *OfHATs* and (**b**) *OfHDACs*. The figure on the left shows the different *cis*-acting elements, which are represented by boxes in different columns. The picture on the right provides the statistics regarding the number of each of the four kinds of *cis*-acting elements in the OfHM promoters.

Promoters are crucial in initiating gene transcription. To explore the potential biological functions and response characteristics of *OfHM* genes, the promoter sequences were submitted to PlantCARE for the analysis of *cis*-acting elements. The analysis revealed 24 *cis*-acting elements involved in light responsiveness, hormone responsiveness (including responses to auxin, GA, SA, MeJA and ABA), physiological stress (such as drought and cold) and growth and development regulation (Figure 4 and Figure 5). The abundance of light response elements was highest at 2259, followed by hormone response (1515), physiological stress elements (917), and growth and development regulation (346) (Figure 4 and Figure 5). Among them, the *OfHAC4* gene contained the fewest light-responsive elements, with only 3, while *OfJMJ17* had the most (32) (Figure 4 and Figure 5). *OfHAC7*, *OfHDA15*, *OfJMJ4*, *OfSDG8*, *OfSDG46* and *OfSDG65* had the fewest hormone-responsive elements (1), while *OfHDT4*, *OfJMJ1* and *OfJMJ10* possessed the most response elements (19–20) (Figure 4 and Figure 5). *OfHAG12*, *OfHAG33*, *OfJMJ6* and *OfSDG12* lacked physiological stress response elements, while the others contained 1–10 stress response elements (Figure 4 and Figure 5). Interestingly, 38 *OfHMs* (18.27%) lacked regulatory *cis*-acting elements for growth and development, 72 genes (34.62%) had only one *cis*-acting element, and 54 *OfHMs* (25.96%) contained two *cis*-acting elements (Figure 4 and Figure 5). These findings collectively indicate that the expression of *OfHM*s may be regulated by various *cis*-acting elements correlated with light responsiveness, hormone responsiveness, physiological stress, and specific growth and development processes.

### 2.5. Chromosomal Distribution and Synteny Analysis of OfHMs

**Figure 6:**
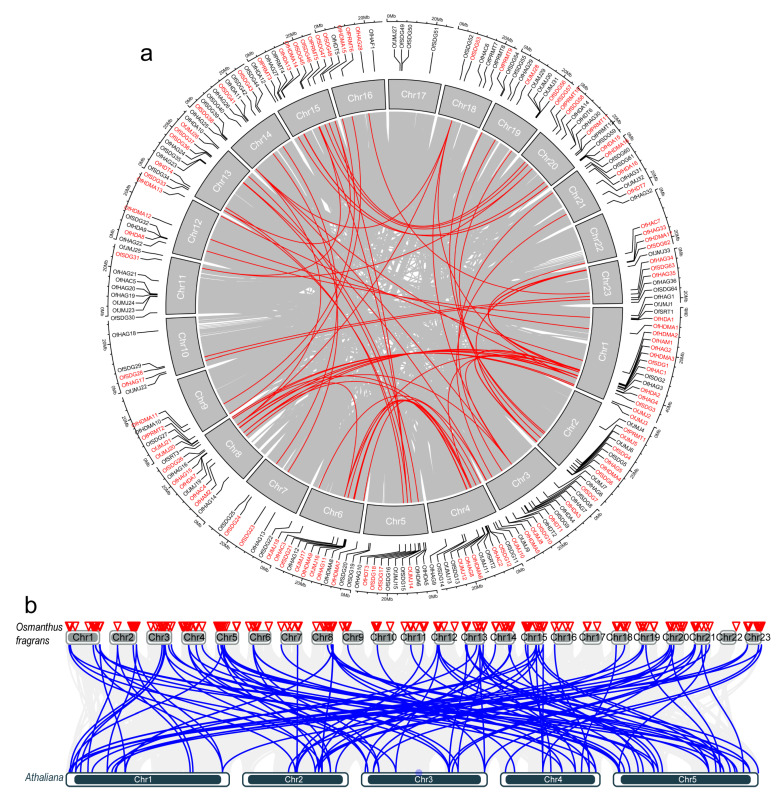
Gene collinearity analysis. (**a**) Synteny of *OfHMs* genes in the *O. fragrans* genome. Colored lines connecting two genes indicate syntenic regions. (**b**) Synteny of *HM* genes between *O. fragrans* and *Arabidopsis* genomes. Colored lines connecting two genes indicate syntenic regions.

The chromosomal distribution of the *OfHM* genes is illustrated in Figure 6a. *OfSDG65*–*69*, *OfJMJ34*, *OfHAG37*–*39* and *OfHDA17* were not anchored on any chromosome (Chr), whereas the remaining 198 *OfHMs* were unevenly distributed across 23 Chrs. The majority of genes were located on Chrs 1 (18 *OfHM* genes), 4 (15), 15 (12), 20 (12), 3 (12), 5 (12), 8 (12), 6 (10), 13 (10) and 14 (10), whereas only one gene was located on Chr 22 (Figure S1).

The importance of gene duplication in the generation of new genes and functions is evident, with segmental and tandem duplications serving as primary drivers during the expansion of gene family [39]. This study investigated the amplification of the *OfHM* genes through an analysis of gene duplication events. The findings revealed that 89 *OfHMs* resulted from duplication events, covering 3 pairs of tandem duplicate genes (i.e., *OfJMJ23* and *OfJMJ24*, *OfSDG49* and *OfSDG50*, and *OfHDT1* and *OfHDT2*) and 86 pairs of segmental duplications, with 70 pairs located on Chrs (Figure 6a). To assess the selective pressure on *OfHM* gene duplication during *O. fragrans* evolution, this study calculated the non-synonymous (Ka)/synonymous (Ks) ratios for homologous gene pairs. Among them, 44 pairs of *OfHM* genes exhibited a Ka/Ks ratio < 1 (Table S2), suggesting purifying selection and evolutionary conservation of functions. On the contrary, 11 pairs of *OfHM* genes, including *OfHDMA1* and *OfHDMA12*, *OfHAC1* and O*fHAC2*, *OfHAC2* and *OfHAC4*, *OfHAC3* and *OfHAC4*, *OfHAC1* and *OfHAC7*, *OfJMJ8* and *OfJMJ18*, *OfJMJ26* and *OfJMJ14*, *OfHAG17* and *OfHAG35*, *OfSDG36* and *OfSDG17*, *OfSDG45* and *OfSDG18*, and *OfSDG57* and *OfSDG65*, displayed a Ka/Ks ratio > 1 (Table S2), indicating positive selection during evolution.

As shown in Figure 6b, a syntenic map of the *OfHMs* and *AtHMs* was constructed to elucidate their potential evolutionary relationships. A total of 87 segmental duplications of *OfHMs* and *AtHMs* were identified (83 pairs of genes shown in Chrs, Figure 6a) (Table S3). The identified pairs consisted of 6 *PRMTs*, 44 *SDGs*, 2 *HDMAs*, 16 *JMJs*, 3 *HAGs*, 1 *SRT*, 9 *HDAs* and 6 *HDTs*. To assess the selection pressure during duplication, we calculated the Ka/Ks values for these gene pairs. It was found that 11 pairs of *HMs*, namely *AtPRMT13* and *OfPRMT5*, *AtPRMT13* and *OfPRMT10*, *AtSDG6* and *OfSDG24*, *AtSDG6* and *OfSDG43*, *AtSDG13* and *OfSDG48*, *AtSDG8* and *OfSDG62*, *AtSDG5* and *OfSDG64*, *AtJMJ28* and *OfJMJ2*, *AtJMJ18* and *OfJMJ3*, *AtJMJ17* and *OfJMJ6*, and *AtJMJ11* and *OfJMJ12*, exhibited Ka/Ks values < 1 (Table S3), indicating purifying selection during evolution. A pair of *HMs*, namely *AtSDG13* and *OfSDG18*, had a Ka/Ks value of 1 (Table S3), suggesting neutral selection during evolution. For 20 pairs of *HMs*, that is, 9 pairs of *SDGs*, 1 pair of *HDMAs*, 7 pairs of *JMJs*, 2 pairs of *HAGs* and 1 pair of *HDAs*, the Ka/Ks value was > 1 (Table S3), indicated that they had undergone positive selection.

### 2.6. Functional Enrichment Analysis of OfHMs

**Figure 7:**
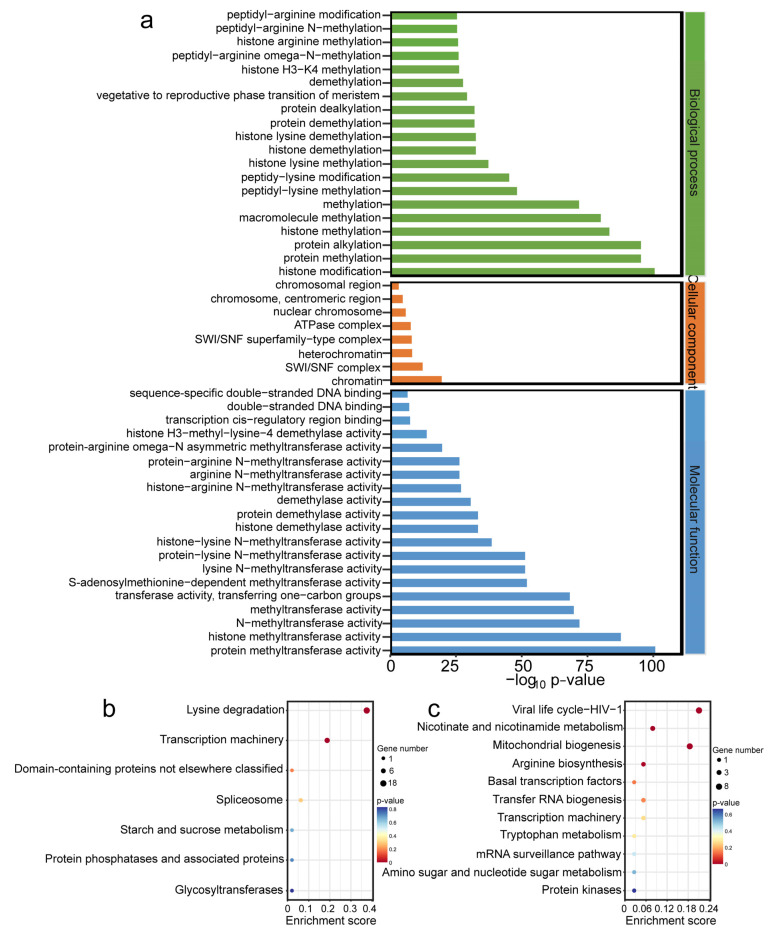
Functional enrichment analysis of *OfHM* genes. (**a**) GO enrichment analysis of histone methylation genes. The top 20 pathways with a gene number greater than 5 are shown. The *x*-axis represents -log10(*p*-value) and the *y*-axis represents the enriched GO terms. (**b**) KEGG analysis of histone methylation genes; (**c**) KEGG analysis of histone acetylation genes. In these analyses, the colors and sizes of the dots correspond to the significance (*p*) and the number of genes, respectively.

An enrichment analysis was performed to uncover the potential biological functions of the *OfHMs.* The functions of all HM-modified genes were determined based on Gene Ontology (GO) categories, encompassing biological processes, molecular functions and cellular components (Figure 7a). In terms of biological processes, these genes exhibited enrichment in pathways such as methylation, macromolecule methylation, HM, protein alkylation, protein methylation and protein methylation. Regarding cellular components, the genes were more abundant in heterochromatin, the SWI/SNF complex and chromatin pathways. For molecular functions, enrichment was observed in pathways such as (N-)methyltransferase activity, histone methyltransferase activity, and protein methyltransferase activity. However, the histone acetylation modification genes did not exhibit enrichment within the relevant metabolic pathways.

Furthermore, analysis of the Kyoto Encyclopedia of Genes and Genomes (KEGG) pathways found that the genes associated with histone methylation modifications are enriched in the lysine degradation and transcription machinery pathways (Figure 7b). On the contrary, the histone acetylation modification genes were more abundant in pathways such as the viral life cycle of HIV-1, the metabolism of nicotinate and nicotinamide, mitochondrial biogenesis, arginine biosynthesis and basal transcription factors (Figure 7c). Consequently, these *OfHM* genes are presumed to play a variety of roles in cellular metabolism.

### 2.7. Prediction of Interactions of OfHM Proteins

**Figure 8:**
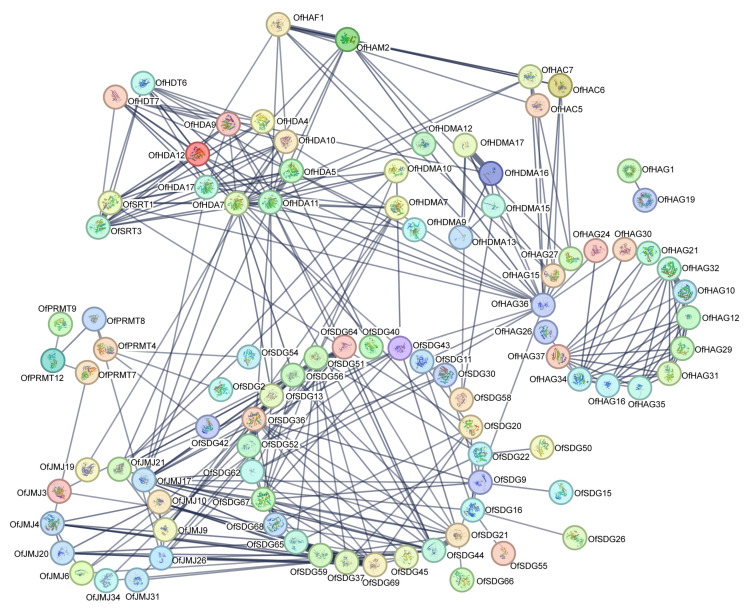
Interaction analysis of OfHM proteins. Circles represent genes, and lines represent *OfHM* gene pairs that may interact.

For a more comprehensive understanding of the biological interactions involving the OfHM proteins, we performed a protein interaction network analysis. As illustrated in Figure 8, 93 proteins from 11 HM-related groups, consisting of 33 OfSDGs, 18 OfHAGs, 12 OfJMJs, 8 OfHDAs, 8 OfHDMAs, 5 OfPRMTs, 3 OfHACs, 2 OfHDTs, 2 OfSRTs, 1 OfHAF and 1 OfHAM, exhibited direct or indirect interactions with other proteins. Among them, the OfSDGs showed the highest number of interactions (202), followed by the OfHAGs (114), OfHDAs (91) and OfJMJs (73). Sixteen genes demonstrated interactions with more than 10 proteins, with OfHDA7 and OfHAG36 showing the most extensive interactions (21), followed by OfHDA11 (19), OfJMJ17 (17), OfSDG67 (15), OfSDG44 (14), OfSDG69 (13) and OfSDG64 (13). In contrast, 14 proteins, including OfHAG1/19/26, OfHDMA12, OfJMJ19, OfPRMT9 and OfSDG2/15/26/42/50/54/55/66, exhibited interactions with only one protein. These findings suggest that *OfHMs* participate in various biological processes by regulating or being regulated by other genes.

### 2.8. Expression Analysis of OfHMs in Different Tissues and Flowering Stages

**Figure 9:**
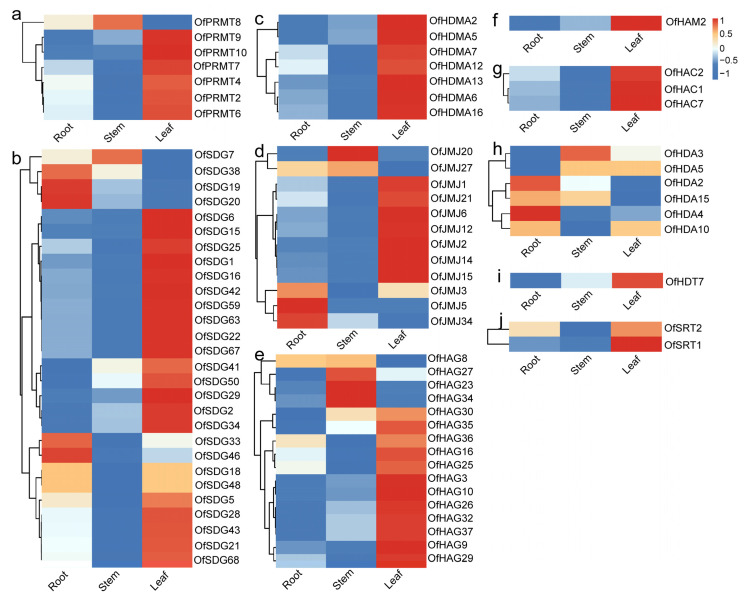
Heatmaps depicting the expression patterns of differentially expressed *OfHMs* in various tissues. (**a**) *OfPRMTs*; (**b**) *OfSDGs*; (**c**) *OfHDMAs*; (**d**) *OfJMJs*; (**e**) *OfHAGs*; (**f**) *OfHAMs*; (**g**) *OfHACs*; (**h**) *OfHDAs*; (**i**) *OfHDTs* (HD2); and (**j**) *OfSRTs* (silent information regulator 2). The differently colored squares represent the genes, with red and blue representing high and low expression levels.

To gain insights into the responsiveness of *OfHMs* during the flower opening and senescence stages, as well as under ethylene and Aza treatments, we examined their expression profiles utilizing publicly available transcriptome data [40]. Among the identified *OfHMs*, 182 genes with expression levels (fragments per kilobase of exon model per million mapped fragments values (FPKM)max > 1) were recognized across various tissues and flowering stages, comprising 11 diverse gene families (Figures S2 and S3). Within this set, 83 *OfHMs*, including *7 OfPRMTs*, 28 *OfSDGs*, 7 *OfHDMAs*, 12 *OfJMJs*, 16 *OfHAG*s, 1 *OfHAM*, 3 *OfHACs*, 6 *OfHDAs*, 1 *OfHDT* and 2 *OfSRTs*, exhibited differential expression in three tissues (roots, stems and leaves) (Figure 9). The majority of *OfHMs* (61 genes, 73.49%), spanning various gene families involving *OfPRMTs* (6, 85.71%), *OfSDGs* (21, 75.00%), *OfHDMAs* (7, 100.00%), *OfJMJs* (7, 58.33%), *OfHAGs* (12, 75.00%), *OfHAM* (1, 100%), *OfHACs* (3, 100%), *OfHDA* (1, 16.67%), *OfHDT* (1, 100%) and *OfSRTs* (2, 100%), exhibited higher expression in leaves, while a small percentage (14.46%) demonstrated elevated expression in roots and 10.84% exhibited increased expression in stems (Figure 9).

**Figure 10:**
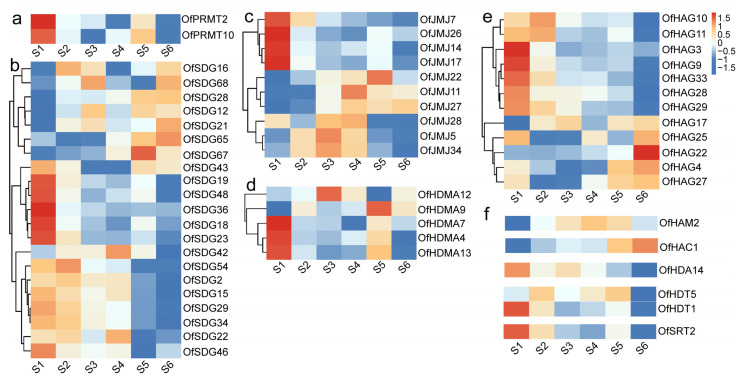
Heatmaps depicting the expression patterns of differentially expressed *OfHMs* during the process of flower opening and senescence. (**a**) *OfPRMTs*; (**b**) *OfSDGs*; (**c**) *OfJMJs*; (**d**) *OfHDMAs*; (**e**) *OfHAGs*; (**f**) *OfHAMs*, *OfHACs*, *OfHDAs*, *OfHDTs* and *OfSRTs*. The differently colored squares within the heatmaps represent the genes, with red and blue representing high and low expression levels.

During the process of flower opening and senescence, 56 differentially expressed *OfHMs* were identified (Figure 10). These genes represented various families, including 2 *OfPRMTs*, 21 *OfSDGs*, 5 *OfHDMAs*, 10 *OfJMJs*, 12 *OfHAGs*, 1 *OfHAM*, 1 *OfHAC*, 1 *OfHDA*, 2 *OfHDTs* and 1 *OfSRT*. Notably, 9 *OfHMs* (16.07%), i.e., *OfSDG12*/*21*/*28*/*68*, *OfHDMA9*, *OfJMJ27*, *OfHAG17*/*22* and *OfHAC1*, exhibited a discernible upward trend, while 20 *OfHMs*, i.e., 8 *OfSDGs*, 2 *OfHDMAs*, 4 *OfJMJs*, 4 *OfHAGs*, 1 *OfHAM* and 1 *OfHDT*, displayed a stable trend. The remaining genes (27, accounting for 48.21%) showed a downward trend (Figure 10). It is noteworthy that the downregulated genes primarily represented eight gene families, namely *OfPRMTs* (2, 100.00%), *OfHDTs* (2, 100.00%), *OfHDA* (1, 100.00%), *OfSRT* (1, 100.00%), *OfJMJs* (5, 50.00%), *OfHAGs* (6, 50.00%), *OfHDMAs* (2, 40.00%) and *OfSDGs* (8, 38.10%) (Figure 10). Among them, the majority of genes (22, 81.48%) exhibited a decrease from S1 (linggeng stage) to S3 (early full flowering stage), an increase in S4 (full flowering stage) or S5 (late full flowering stage), and a subsequent decrease in S6 (abscission stage) (Figure 10).

### 2.9. Expression Analysis of OfHMs under Ethylene and 5′-Azacytidine (Aza) Treatment

**Figure 11:**
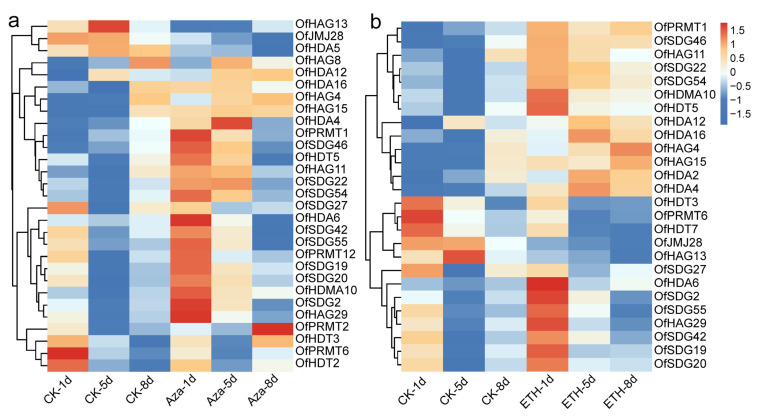
Heatmaps depicting the expression patterns of differentially expressed *OfHMs* in response to Aza or ethylene treatment. (**a**) After Aza treatment; (**b**) after ethylene treatment. The differently colored squares represent the genes, with red and blue representing high and low expression levels.

In response to Aza or ethylene treatment, 132 *OfHMs* were expressed (FPKMmax > 1) (Table S4). Among them, 29 *OfHMs*, i.e., 4 *OfPRMTs*, 9 *OfSDGs*, 1 *OfHDMA*, 1 *OfJMJ*, 6 *OfHAGs*, 5 *OfHDAs* and 3 *OfHDTs*, were differentially expressed after Aza treatment (Figure 11a). Specifically, 22 genes (75.86%), including 1 *OfHDMA* (100.00%), 5 *OfHAGs* (83.33%), 4 *OfHDAs* (80.00%), 7 *OfSDGs* (77.78%), 3 *OfPRMTs* (75.00%) and 2 *OfHDTs* (66.67%), displayed upregulated expression after treatment, while 5 genes, comprising *OfSDG42*, *OfJMJ28*, *OfHAG13*, *OfHDA5* and *OfHDT3*, showed downregulated expression. Furthermore, 26 *OfHMs*, i.e., 2 *OfPRMTs*, 9 *OfSDGs*, 1 *OfHDMA*, 1 *OfJMJ*, 5 *OfHAGs*, 5 *OfHDAs* and 3 *OfHDTs*, were differentially expressed after ethylene treatment (Figure 11b). Among them, 21 genes (80.77%), including 9 *OfSDGs* (100.00%), 5 *OfHDAs* (100.00%), 1 *OfPRMT* (50.00%), 1 *OfHDMA* (100.00%), 4 *OfHAGs* (80.00%) and 1 *OfHDT* (33.33%), demonstrated upregulated expression after treatment. Additionally, 18.52% of the genes, comprising *OfPRMT6*, *OfJMJ28*, *OfHAG13*, *OfHDT3* and *OfHDT7*, exhibited downregulated expression after treatment. These findings indicate a crucial role of *OfHMs* in response to ethylene and Aza treatments, as well as in the processes of flower opening and senescence in *O. fragrans*.

### 2.10. Quantitative Real-Time Polymerase Chain Reaction (qRT-PCR) Analysis of OfHM Genes

**Figure 12:**
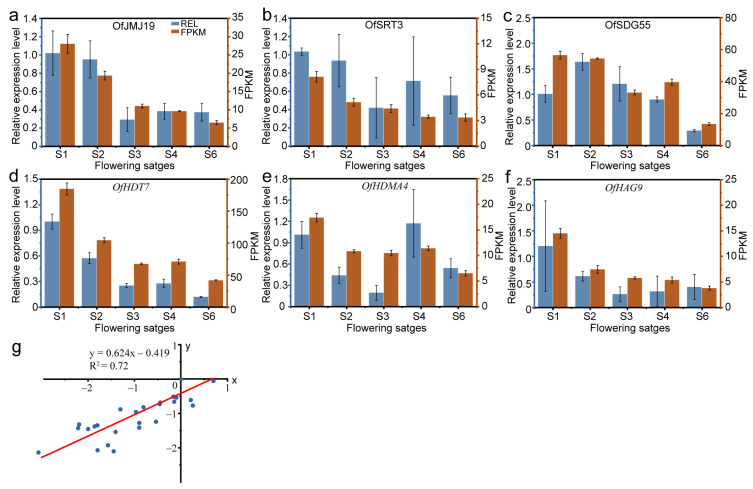
The expression levels of *OfHMs* were validated utilizing quantitative real-time polymerase chain reaction (qRT-PCR). (**a**) *OfJMJ19*; (**b**) *OfSRT3*; (**c**) *OfSDG55*; (**d**) *OfHDT7*; (**e**) *OfHDMA4*; and (**f**) *OfHAG9*. The *x*-axis represents the different flowering stages, including S1, S2, S3, S4 and S6. The right and left *y*-axes represent the fragments per kilobase of exon model per million mapped fragments (FPKM) values and the relative expression levels (REL), respectively. The values are presented as the mean ± standard deviation. (**g**) A linear fitting analysis was performed on the qRT-PCR and FPKM data. The *x*-axis represents the qRT-PCR data, and the *y*-axis represents the FPKM data.

To validate the reliability of the *OfHM* expression profiles derived from the RNA-seq data, we determined the expression levels of six *OfHMs* during the flower opening and senescence stages using qRT-PCR. The results revealed that the expression patterns of these *OfHMs* closely resembled those obtained in the RNA-seq analysis (Figure 12). Specifically, the expression of these genes, *OfJMJ19*, *OfSRT3*, *OfSDG55*, *OfHDT7*, *OfHDMA*4 and *OfHAG9*, showed a decreasing trend during flower opening and senescence (Figure 12a–f). Furthermore, the R^2^ value reached 0.72 through linear fitting between the qRT-PCR and FPKM data (Figure 12g), indicating a high degree of reliability in the *OfHM* gene profiles. Therefore, our transcriptome-based analysis of *OfHM* gene expression levels demonstrates high reproducibility and reliability, providing a robust reference for further studying the roles of *OfHMs*.

## 3. Discussion

Numerous previous studies have highlighted the pivotal role of *HMs* in plant growth and development processes, along with responses to both abiotic and biotic stresses, through the nuanced modulation of gene transcription [14,16]. Consequently, an increasing number of investigations have focused on understanding the functions of modifying enzymes in various plant species. Although substantial progress has been made in some model plants [7,8,16], this information has not been reported for *O. fragrans* until now. Here, we conducted a comprehensive characterization of *OfHMs*, providing insights into their gene location, conserved domains, phylogenetic relationships, gene expansion, *cis*-acting elements and gene structure. Furthermore, we analyzed the expression patterns of *OfHMs* during flower opening and senescence, and under stress treatments. The findings presented here contribute to the growing knowledge in this field and provide a basis for further research on *O. fragrans*.

### 3.1. O. fragrans HM Genes in Comparison with Other Plant Species

Here, we identified 208 *OfHMs* (Table S1). These included 81 *OfHMTs* (comprising 12 *OfPRMT*s and 69 *OfSDGs*), 51 *OfHDMs* (encompassing 17 *OfHDMAs* and 34 *OfJMJs*), 49 *OfHATs* (including 39 *OfHAGs*, 2 *OfHAMs*, 7 *OfHACs* and 1 *OfHAF*) and 27 *OfHDACs* (involving 17 *OfHDAs*, 3 *OfSRTs* and 7 *OfHDTs*) (Table S1). Notably, the number of *OfHMs* is comparable to that observed in other plants, such as *MdHMs* (198) [8,41], and 1.53 to 2.39-fold higher compared to *SlHMs* (124) [14], *LcHMs* (87) [15], *CsHMs* (136) [16] and *AtHMs* (102) [7] (Table S1). This observation suggests that the level of duplication of *HM* genes is higher during the evolutionary process of *O. fragrans*.

The uneven distribution of *OfHMs* in the genomes, as observed in Figure 6a, aligns with findings in citrus [16] and apple [8]. This pattern of uneven chromosome distribution suggests that these changes occurred prior to species differentiation.

### 3.2. Evolution and Amplification Analyses of OfHM Gene Family

The construction of phylogenetic trees, aligning genes across diverse botanical species, provides profound insights into the intricate tapestry of evolutionary interrelations among genes [42]. In this study, four phylogenetic trees, i.e., HMTs, HDMs, HATs and HDACs, were constructed, utilizing all members of the *HM* gene families from both *O. fragrans* and *A. thaliana* (Figure 1). The observed clustering pattern for each category of *OfHMs* is consistent with observations documented in other species, as reported in previous studies [7,8,14,17]. However, there are exceptions, exemplified by the clustering of HDAs on a particular branch, excluding AtHDT4 (Figure 1d). This divergence might be attributed to the partial matching of their protein sequences.

The structure of genes and conserved motifs are pivotal in shaping the evolution of gene families. In our study, different gene families within the OfHMs showcased conserved typical domains, as illustrated in Figure 2 and Figure 3 [7,8,14,17]. Notable examples include the presence of a PRMT5/MT domain in the OfPRMTs (Figure 2a), a characteristic SET domain in the OfSDGs (Figure 2b), and cupin_RmlC-like superfamily/JmjC and SWIRM conserved domains in the OfJMJs and OfHDMAs, respectively (Figure 2c). Furthermore, the domains AT, HAT_KAT11 and MOZ-SAS were identified in the OfHAGs, OfHACs and OfHAMs (Figure 3a,b), while the HDAC1, SIR2 and NPL domains were contained in the OfHDAs, OfSRTs and OfHDTs, respectively (Figure 3c). Additionally, various genes within each gene family harbored additional structural domains (Figure 2 and Figure 3). For instance, the I-type OfSDGs featured SET domains; the II-type OfSDGs included AWS domains; the III-type OfSDGs, excluding OfSDG5 (9), encompassed zf-HC5HC2H and FYRC/PWWP domains; and the V-type OfSDGs typically contained SRR/WIYLD and Pre-SET domains; and most of the VI/VII-type OfSDGs included rubis-subs-bind/AWS/TPR domains (Figure 2b). This conservation of domains within the same family/subfamily implies shared features across species. Furthermore, OfHMs with distinct structures and conserved motifs exhibited clustering at considerable distances, whereas those with analogous structures and motifs tended to cluster close together (Figure 2 and Figure 3). This clustering pattern is consistent with the observations in other species [8,16], indicating a potential correlation among phylogeny, gene structure and protein motifs. However, these correlations require validation through additional experiments.

Segmental and tandem duplications serve as fundamental mechanisms driving the expansion of gene families [39]. In our study, 89 *OfHMs* were identified as products of duplication events, and 86 *HM* segmental duplications existed between the *OfHMs* and *AtHMs* (Figure 6b). This discovery aligns with results in apple [8,41], where 67 pairs of *MdHMs* were reported. Most of the *OfHM* gene pairs exhibited a Ka/Ks ratio < 1 (Table S2), indicative of purifying selection and evolutionary conservation of their functions. In particular, 11 *OfHM* gene pairs, including 1 *OfHDMA*, 4 *OfHACs*, 2 *OfJMJs*, 1 *OfHAG* and 3 *OfSDGs*, exhibited a Ka/Ks ratio > 1 (Table S2). However, the majority of pairs of *AtHM* and *OfHM* genes displayed a Ka/Ks ratio > 1 (Table S3), indicating positive selection during evolution [43]. These results diverged from most studies [8,17,41]. For instance, gene pairs duplicated within apple, millet (*Setaria italica*), or between them and *Arabidopsis* generally exhibited Ka/Ks ratios < 1 (Table S3). These gene pairs with Ka/Ks ratios greater than 1 may have played a pivotal role in species adaptive evolution or in response to specific environmental stresses. Collectively, the *OfHMs* have undergone tandem and segmental duplications, contributing significantly to the expansion of the *OfHMs* and their subsequent structural and functional diversification.

### 3.3. OfHMs Are Involved in the Flowering Process and the Induction of Aza and Ethylene Responses

*HM* genes play a crucial role in the orchestration of plant growth and development [14,16]. The functional enrichment analysis revealed that *OfHMs* were enriched in lysine degradation, transcription machinery, viral life cycle of HIV-1, nicotinate and nicotinamide metabolism, mitochondrial biogenesis, arginine biosynthesis and basal transcription factors pathways (Figure 7b,c). The promoter region of the *OfHM* genes contained elements that respond to plant hormones, stress, light, and growth and development (Figure 4 and Figure 5), indicating their involvement in light responses, hormone responses, physiological stress, and the regulation of growth and development.

The expression patterns of genes provide vital information for the exploration of gene function [44,45]. Across three different tissues (roots, stems and leaves), 83 *OfHMs* exhibited differential expression patterns, with the majority of genes (73.49%) exhibiting increased expression in leaves (Figure 9). However, it should be noted that certain studies have observed nearly ubiquitous expression of *TaHMs* during the development stages of wheat grains, with numerous genes displaying elevated expression levels in specific layers of grain tissue [46], thus indicating potential species-specific differences.

In total, 56 *OfHMs* exhibited differential expression during the flower opening and senescence stages, with 27 genes, including *OfPRMTs* (2, 100.00%), *OfHDTs* (2, 100.00%), *OfHDA* (1, 100.00%), *OfSRT* (1, 100.00%), *OfJMJs* (5, 50.00%) and *OfHAGs* (6, 50.00%), *OfHDMAs* (2, 40.00%) and *OfSDGs* (8, 38.10%), downregulated or showing a down (S1–S3)–up (S4 or S5)–downregulated (S6) pattern (Figure 10). In *Arabidopsis* and rice, genes such as *AtPRMT5* [47], *AtHDMA* [48], *OfHDT1* [49], *OfSRT1* [50], *AtSDG* [33,51,52,53], *AtHDA* [26,48,54] and *AtJMJ* [41,55] participate in flower development. It is noteworthy that genes within the same subfamily may exert different effects. For instance, members of Class III, such as *AtJMJ27* and *AtJMJ28*, have been demonstrated to participate in the regulation of plant flowering. Specifically, *AtJMJ27* inhibits flowering by directly or indirectly modulating the modification of H3K9me2 at the *FLC* and CONSTANS (*CO*) gene loci [56]. Conversely, *AtJMJ28* interacts with FLOWERING BHLH (*FBH*) and affects flowering by removing H3K9me2, thereby activating *CO* expression [57]. Consequently, we hypothesize that the downregulated expression of *OfPRMTs*, *OfHDTs*, *OfHDAs* and *OfSRTs* promotes flower opening and senescence, while upregulated or downregulated expression of *OfSDGs*, *OfHDMAs*, *OfJMJs* and *OfHAGs* regulates flower opening and senescence. However, the specific roles of individual genes require further analysis.

After Aza treatment, the expression of 29 *OfHMs* showed significant differences, with 22 genes (1 *OfHDMA* (100.00%), 5 *OfHAGs* (83.33%), 4 *OfHDAs* (80.00%), 7 *OfSDGs* (77.78%), 3 *OfPRMTs* (75.00%) and 2 *OfHDT* (66.67%)) exhibiting upregulated expression (Figure 11a). Similarly, after ethylene treatment, 26 *OfHMs* exhibited significant differential expression, with 21 genes (9 *OfSDGs* (100.00%), 5 *OfHDAs* (100.00%), 1 *OfPRMT* (50.00%), 1 *OfHDMA* (100.00%), 4 *OfHAGs* (80.00%) and 1 *OfHDT* (33.33%)) displaying upregulated expression (Figure 11b). There are limited studies that have reported on the impact of Aza and ethylene treatments on *HMs*. In our previous studies, we observed that Aza treatment accelerated the flowering process of *O. fragrans*, inducing a change in flower color from yellow to orange [40]. This suggests that the DNA methylation inhibitor Aza induces ethylene synthesis, triggering premature opening and senescence phenotypes. The plant hormone ethylene influences various aspects of plant growth and development, including certain forms of programmed senescence, such as promoting flower opening and senescence [58,59]. Therefore, we hypothesize that the Aza or ethylene treatment can promote the expression of genes such as *OfHAGs, OfHDAs* and *OfSDGs* (Figure 11), thereby promoting the blooming and aging of *O. fragrans*. For example, *AtJMJ12* can bind to regulatory genes of senescence, such as ETHYLENE INSENSITIVE 2 (*EIN2*), oxygen responsive element 1 (*ORE1*), NAM, ATAF1, 2, CUC2 (*NAC3*), NAC-like (*NAP*) and non-yellowing 1/2 (*NYE1/2*), and catalyze the demethylation of H3K27me3 in the promoter/CDS to activate gene expression and accelerate leaf senescence [60]. *AtHDA5* forms a repressor complex with *AtHDA6*, *FVE* and *FLD*, which inhibits *FLC* and *MAF1,2,4* (affecting flowering genes) through deacetylation of H3K9 and H3K14 lysine residues, playing a vital role in the control of flowering in *Arabidopsis* [26,48,54,61], especially in promoting flowering [61]. It is important to note that our results cannot conclusively identify which *OfHMs* are most closely associated with flower opening and senescence. Determining the core *HMs* involved in these processes requires further research for confirmation.

## 4. Materials and Methods

### 4.1. Identification and Chromosome Location of OfHM Gene Family

To identify members of the *HM* gene family in *O. fragrans*, various genomic data, including whole-genome data, CDS data, protein sequences and the General Feature Format version 3 (GFF3) file, were obtained from the National Center for Biotechnology Information (NCBI) database (PRJNA679852) [62]. Two methods were employed to identify members within the *OfHM* gene family. Firstly, hidden Markov Model (HMMER) files corresponding to specific domains (PF00583, 00850, 00856, 01853, 02146, 02373, 04433, 05185, 08214 and 09247) were acquired from the Pfam database (http://pfam.sanger.ac.uk/, accessed on 25 December 2023), following the approach of previous studies [14,16]. Additionally, an HMMER file was constructed using protein sequences encoded by four *AtHDT* genes, i.e., *AtHDT1*–*4* (*At3g44750*, *At5g22650*, *At5g03740* and *At2g27840*), obtained from the TAIR database (http://www.arabidopsis.org/, accessed on 25 December 2023). Subsequently, these 11 HMMER files served as queries to search the *O. fragrans* genome using HMMER3.0, with an E-value threshold set to <0.00001 [63]. Secondly, a total of 102 AtHM protein sequences [7] were retrieved from the TAIR website. A BLASTP alignment search was performed using these *AtHM* sequences as query sequences, with an expected value (E value) set at 0.00001 to identify candidate *OfHM*s. After eliminating redundant sequences, all candidate protein sequences were analyzed through the SMART database (http://smart.embl.de/, accessed on 28 December 2023) and the Conserved Domain Search (CDD) database (https://www.ncbi.nlm.nih.gov/Structure/bwrpsb/bwrpsb.cgi, accessed on 28 December 2023). Throughout this process, genes lacking known conserved domains were systematically excluded, resulting in the final identification of the *OfHM* genes. The *OfHM* genes, including *HMTs* (*SDGs* and *PRMTs*), *HDMs* (*HDMAs* and *JMJs*), *HATs* (*HAGs*, *HAMs*, *HACs* and *HAFs*) and *HDACs* (*HDAs*, *SRTs* and *HDTs*), were named according to their Chr orders, following the convention of a previous study [16].

The chromosomal location information for the *OfHM* genes were extracted from the *O. fragrans* genome, and the MG2C online tool (http://mg2c.iask.in/mg2c_v2.1/, accessed on 31 December 2023) was utilized to visually represent their chromosomal locations.

### 4.2. Physicochemical Characteristics Examination, Phylogenetic Tree Elaboration, and Gene Structure Research

ProtParam (https://web.expasy.org/protparam/, accessed on 13 January 2024) was employed for the analysis of the physical and chemical properties of proteins. Deep TMHMM (https://dtu.biolib.com/DeepTMHMM, accessed on 12 January 2024) was used to predict the transmembrane structure of the OfHM proteins. Additionally, the BUSCA website (http://busca.biocomp.unibo.it/, accessed on 13 January 2024) was used to predict their subcellular localization.

In conducting the phylogenetic analysis, molecular evolutionary genetics analysis (MEGA) *v*11.0 [64] was used to explore the phylogenetic relationships of *HMs* between *O. fragrans* and *A. thaliana*. The protein sequences of AtHMs and OfHMs were aligned by the MUSCLE program with default parameters. Subsequently, the resulting multiple sequence alignment files were utilized to construct phylogenetic trees with the neighbor-joining (NJ) or maximum likelihood method, with bootstrap values determined through 1000 times.

The TBtools software v2.061 [65] was employed to determine the positions of untranslated regions (UTRs), introns, CDSs and domains within the *O. fragrans* genome annotation file (gff3), and to visualize the gene structure. For the prediction of conserved motifs in *OfHMs*, the MEME website (https://meme-suite.org/meme/tools/meme, accessed on 30 December 2023) was utilized with the specified parameter motif = 20 and any number of repetitions in the site distribution. Subsequently, the identified motifs were visualized using the TBtools software [65]. The CDD database was employed to predict the conserved domains of *OfHMs*, and the results were visualized using the TBtools software [65].

### 4.3. Cis-Acting Regulatory Element Analysis of OfHM Genes

The 2000 bp base sequence upstream of the start codon (ATP) for each member of the *HM* gene family was extracted as the promoter region using TBtools [65]. Subsequently, Plant CARE (http://bioinformatics.psb.ugent. be/webtools/plantcare/html/, accessed on 1 January 2024) [66] was employed to predict *cis*-acting regulatory elements within these promoter regions. The identified *cis*-acting regulatory elements were then visualized using the TBtools software [65].

### 4.4. Tandem Duplication and Synteny Analyses

Tandem duplication and synteny relationships were explored. Tandem duplication of *OfHM*s genes was identified according to their physical locations in individual Chrs within the *O. fragrans* genome. Specifically, genes located within a 200 kb region on the Chr, exhibiting > 70% identity, were defined as tandem duplication genes [39]. Furthermore, synteny blocks among different *O. fragrans* Chrs were identified using the multiple collinearity scan (MCScanX) toolkit [67].

The *Arabidopsis* genome was obtained from the Phytozome *v*13 database (https://phytozome-next.jgi.doe.gov/info/Athaliana_Araport11, accessed on 14 April 2023). Collinearity maps of *HM* genes between the *Arabidopsis* and *O. fragrans* genomes were constructed using TBtools [65]. Additionally, DnaSP *v*5 [68] was utilized to calculate the Ka/Ks ratio at each site.

### 4.5. Protein–Protein Interaction Network Construction and Functional Enrichment Analysis of OfHM Genes

A BLASTP analysis was utilized to identify the homologous genes of *OfHM*s and *AtHM*s. Interaction pairs among the *OfHMs* were determined using the STRING database *v*12.0 (http://string-db.org, accessed on 21 January 2024). For this analysis, the network type was configured as the full STRING network, where the edges represent both functional and physical protein associations; the meaning of network edges was adjusted to reflect the confidence in thickness, indicative of the strength of the data support; and a minimum required interaction score of high confidence (>0.70) was applied to ensure the reliability of the identified interactions. The visualization of gene pairs with high confidence scores was achieved using Cytoscape *v*3.6.1 (http://www.cytoscape.org/, accessed on 22 January 2024). Furthermore, GO and KEGG enrichment analyses of histone methylation and acetylation genes were performed using the resources available at https://www.omicsshare.com/(accessed on 22 January 2024), respectively.

### 4.6. Plant Materials and Treatment

The experimental material for this study was a healthy and well-growing *O. fragrans* ‘Liuyejingui’ (OFL) tree located on the campus of Huazhong Agricultural University in Wuhan, Hubei, China (114°21′ W, 30°29′ N), as detailed by Chen et al. [62]. Various tissues, including roots, stems and leaves, were collected from the same tree, alongside samples from different stages of flowering (S1: linggeng stage, characterized by closed flower buds; S2: initial flowering stage, where flowers exhibit a slight opening with a petal angle of less than 45°; S3: early stage of full flowering, with flowers partially open and petal angles ranging from 45 to 90°; S4: full stage of flowering, where petals are fully expanded; S5: late stage of full flowering, marked by petals slightly losing turgor and darker pollen color; and S6: stage of abscission, during which petals lose turgor and undergo natural abscission) [62]. Detached branches that carried floral buds at stage S1 were treated with 200 mL of 10 mM 5′-azacytidine (Aza) or 500 mg L^−1^ ethephon (dissolved in ddH_2_O). In contrast, as a control, flowering branches were sprayed with 200 mL of ddH_2_O alone [40]. The spray treatment was repeated at 3-day intervals, specifically targeting flowers at stages S1, S3 and S5. Three independent biological replicates were performed for each treatment.

### 4.7. Identification of OfHM Expression Profiles Using High-Throughput Sequencing

Total RNA was extracted using TRIzol reagent (Invitrogen Co., Carlsbad, CA, USA) following the manufacturer’s protocol. RNA-seq libraries were prepared using the SMARTer cDNA synthesis kit (Clontech Laboratories, Mountain View, CA, USA), and subsequently processed on the MGI-SEQ 2000 platform (Frasergen Bioinformatics Co., Ltd., Wuhan, Hubei, China). Genes with FPKMmax values greater than 1 were selected from the *O. fragrans* samples. The FPKM data were then converted into TPM (transcripts per kilobase of exon model per million mapped reads) data, and the limma package [69] was applied to screen for differentially expressed genes (DEGs) between groups. The default filtering threshold for DEGs was set at |log2FoldChange| > 1 and q-value < 0.05. The generated heatmaps were visualized using the heatmap tool on https://cloud.oebiotech.com (accessed on 31 January 2024). This visualization process involved Z-score normalization and clustering of the row data, ensuring an accurate representation of the underlying patterns and trends within the dataset.

### 4.8. qRT-PCR Analysis of OfHM Genes

A total of 6 *OfHMs*, selected for their high expression from different gene families, underwent assessment of their expression levels at various stages of flowering using qRT-PCR. The qRT-PCR was performed using the Applied Biosystems 7500 Sequence Detection System (ABI7500; Thermo Fisher Scientific, Inc., Waltham, MA, USA). The primers sequences for qRT-PCR were designed using Prime Premier 5 (Table S5). The qRT-PCR reaction mixture, with a total volume of 15 μL, included 2 μL of cDNA, 0.8 μL of each forward and reverse primers, 10 μL of SYBR Mix and 6.4 μL of ddH_2_O. The expression level of *OfRAN1* served as the reference, and qRT-PCR amplification was carried out under the following conditions: 94 °C for 30 s, followed by 40 cycles of 94 °C for 10 s and 60 °C for 30 s. Gene relative expression levels were calculated using the 2^−ΔΔCT^ method [70], with each analysis comprising four replicates. The normalized log2 fold change for each gene was calculated using both RNA-seq and qRT-PCR data, with S1 data serving as the reference. Subsequently, linear fitting was applied to the RNA-seq and qRT-PCR datasets, and the correlation index (R^2^) was computed.

## 5. Conclusions

In this study, we systematically identified *HM* genes within the *O. fragrans* genome, providing comprehensive insights into their chromosomal location, gene structure, phylogenetic relationships and protein–protein interactions. Utilizing high-throughput RNA-seq data, we examined the expression patterns of these genes across the flower opening and senescence stages, various tissues, and in response to specific treatments. The comprehensive findings derived from our research offer a thorough understanding of *OfHMs*, contributing not only to the study of biological theory, but also laying the groundwork for future in-depth analyses.

## Data Availability

Data are contained within the article and Supplementary Materials.

## Supplementary Materials

The following supporting information can be downloaded at: https://www.mdpi.com/article/10.3390/plants13060777/s1, Figure S1: The chromosomal localization of histone modification genes in *Osmanthus fragrans*; Figure S2: Heatmaps illustrating the expression of histone methylation genes from *O. fragrans* across various tissues and flowering stages; Figure S3: Heatmaps illustrating the expression of histone acetylation genes from *O. fragrans* across various tissues and the flowering stages; Table S1: Detailed information and physicochemical properties of *OfHMs*; Table S2: The Ka/Ks values of segmental and tandem duplications between *OfHMs*; Table S3: The Ka/Ks values of segmental duplications between *OfHMs* and *AtHMs*; Table S4: Differentially expressed *OfHMs* detected under Aza or ethylene treatment; Table S5: Gene primers used in the study.
