# Supplementary material for: Genome-Wide Identification of Osmanthus fragrans Histone Modification Genes and Analysis of Their Expression during the Flowering Process and under Azacytidine and Ethylene Treatments
Source: Plants (Basel). 2024 Mar 9;13(6):777. doi: 10.3390/plants13060777 (PMC10976118; doi:10.3390/plants13060777)
Supplement: Supplementary file 1 [file plants-13-00777-s001.zip › plants-2891717-supplementary.pdf]

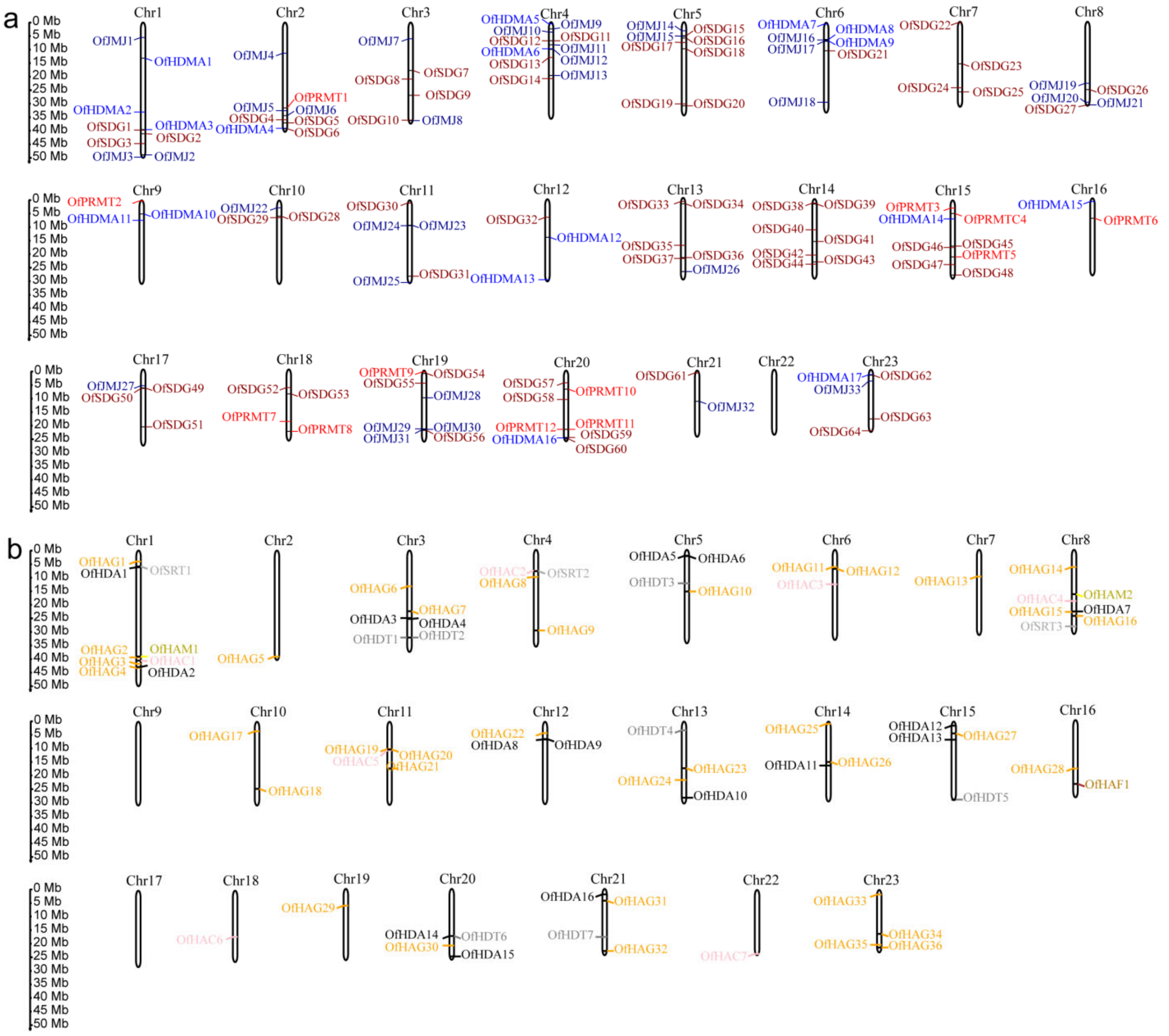

Figure S1. The chromosomal localization of histone modification genes in *Osmanthus fragrans*. (**a**) Histone methylation modification genes; (**b**) histone acetylation modification genes. Different colors represent genes belonging to distinct gene families.

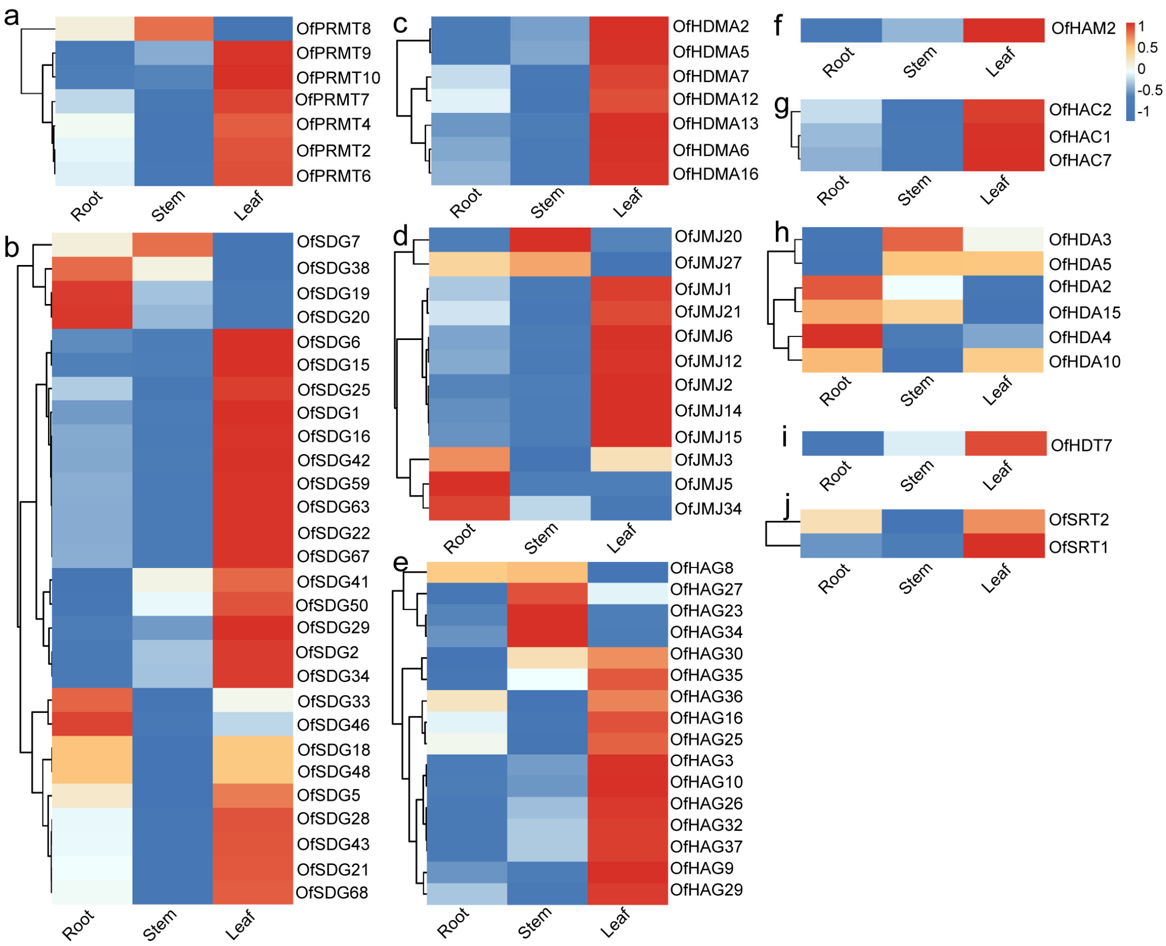

Figure S2. Heatmaps illustrating the expression of histone methylation genes from *O. fragrans* across various tissues and flowering stages. (**a**) *OfPRMTs*; (**b**) *OfSDGs*; (**c**) *OfHDMAs*; and (**d**) *OfJMJs*. The differently colored squares represent the genes, with red and blue representing high and low expression levels, respectively.

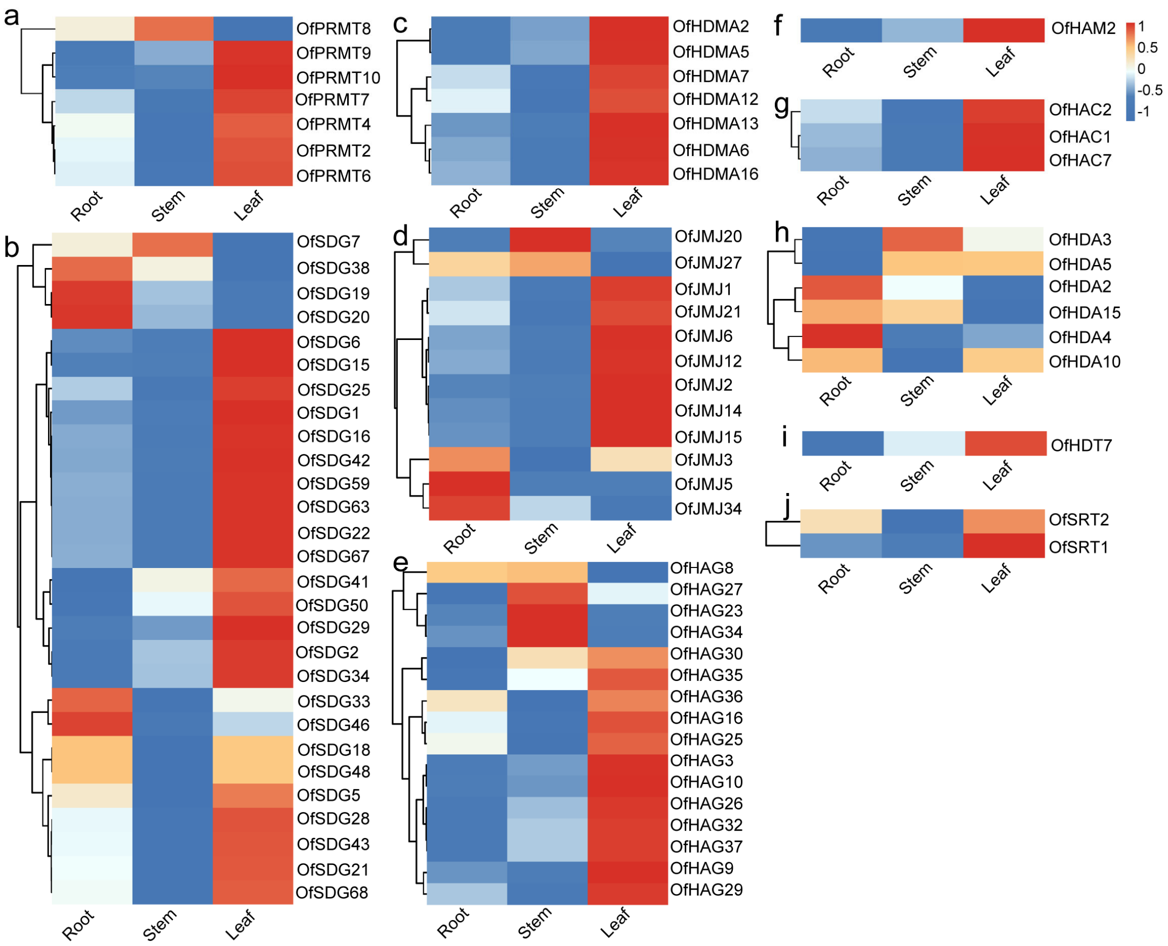

Figure S3. Heatmaps illustrating the expression of histone acetylation genes from *O. fragrans* across various tissues and the flowering stages. (**a**) *OfHAGs*; (**b**) *OfHAMs*; (**c**) *OfHACs*; (**d**) *OfHAFs*; (**e**) *OfHDAs*; (**f**) *OfHDTs*; and (**g**) *OfHAFs*. The differently colored squares represent the genes, with red and blue representing high and low expression levels, respectively.

Table S1. Detailed information and physicochemical properties of *OfHMs*.

Gene name	Gene ID	Chromosome location	Subcellular localization	Number of aa	MW [1]	pI [2]	II [3]	Aliphatic indices	GRAVY [4]	TMR [5]
OfPRMT1	LYG004534	Chr2	endomembrane system	431	48.39	5.55	46.25	72.81	–0.40	0
OfPRMT2	LYG016499	Chr9	nucleus	588	66.17	4.96	51.96	84.00	–0.35	0
OfPRMT3	LYG026685	Chr15	nucleus	305	34.87	5.56	36.85	86.30	–0.35	0
OfPRMT4	LYG026793	Chr15	mitochondrion	755	84.85	5.74	49.26	86.81	–0.21	0
OfPRMT5	LYG027421	Chr15	endomembrane system	537	60.33	5.32	50.82	79.35	–0.32	0
OfPRMT6	LYG028620	Chr16	nucleus	632	70.88	4.55	46.75	77.91	–0.32	0
OfPRMT7	LYG031981	Chr18	plasma membrane	390	44.30	4.81	37.68	81.97	–0.26	0
OfPRMT8	LYG032132	Chr18	plasma membrane	375	42.47	5.56	38.12	80.05	–0.26	0
OfPRMT9	LYG032352	Chr19	plasma membrane	442	49.70	5.46	44.35	71.20	–0.42	0
OfPRMT10	LYG034052	Chr20	endomembrane system	537	60.29	5.48	48.77	81.88	–0.28	0
OfPRMT11	LYG034650	Chr20	endomembrane system	275	31.19	6.99	32.64	95.31	0.02	0
OfPRMT12	LYG034653	Chr20	nucleus	642	72.41	5.49	37.75	88.99	–0.19	0
OfSDG1	LYG002088	Chr1	nucleus	1508	167.08	6.66	47.76	67.39	–0.62	0
OfSDG2	LYG002189	Chr1	chloroplast thylakoid lumen	504	57.63	8.76	43.93	89.23	–0.29	0
OfSDG3	LYG002517	Chr1	nucleus	726	80.16	7.47	44.56	70.96	–0.51	0
OfSDG4	LYG004832	Chr2	nucleus	678	75.35	8.92	33.86	73.88	–0.48	0
OfSDG5	LYG004935	Chr2	nucleus	855	93.50	6.13	48.29	78.05	–0.49	0
OfSDG6	LYG005144	Chr2	nucleus	1485	165.89	8.40	44.50	67.41	–0.73	0
OfSDG7	LYG006022	Chr3	nucleus	709	77.85	7.73	47.62	68.18	–0.47	0
OfSDG8	LYG006170	Chr3	extracellular space	276	30.34	8.52	50.56	87.07	–0.03	0
OfSDG9	LYG006556	Chr3	nucleus	496	54.89	5.35	36.57	67.68	–0.54	0
OfSDG10	LYG007286	Chr3	nucleus	949	106.79	8.38	47.59	70.26	–0.59	0
OfSDG11	LYG008012	Chr4	nucleus	467	53.45	5.14	50.42	92.03	–0.03	0
OfSDG12	LYG008021	Chr4	nucleus	2419	276.20	6.34	52.19	62.62	–0.88	0
OfSDG13	LYG008422	Chr4	nucleus	861	96.62	7.82	45.95	69.02	–0.62	0
OfSDG14	LYG008775	Chr4	nucleus	685	77.19	6.16	40.30	73.99	–0.52	0
OfSDG15	LYG009850	Chr5	chloroplast	467	53.06	6.71	50.53	89.40	–0.28	0
OfSDG16	LYG009920	Chr5	endomembrane system	641	71.74	6.05	42.29	92.84	–0.19	0
OfSDG17	LYG010073	Chr5	nucleus	349	39.80	5.28	40.20	78.22	–0.54	0
OfSDG18	LYG010290	Chr5	nucleus	701	79.63	5.73	57.00	71.18	–0.73	0
OfSDG19	LYG011257	Chr5	endomembr	403	44.80	4.84	60.29	69.75	–0.39	0

			ane system							
OfSDG20	LYG011290	Chr5	endomembrane system	404	44.98	4.91	63.08	71.98	–0.33	0
OfSDG21	LYG012303	Chr6	nucleus	2271	258.79	6.26	52.87	65.76	–0.80	0
OfSDG22	LYG013340	Chr7	organelle membrane	344	38.64	9.01	56.26	84.83	–0.09	0
OfSDG23	LYG014355	Chr7	organelle membrane	688	77.02	5.40	54.26	93.14	–0.15	0
OfSDG24	LYG014656	Chr7	nucleus	1522	170.86	6.14	44.59	83.14	–0.34	0
OfSDG25	LYG014706	Chr7	chloroplast	840	93.46	8.93	53.18	72.73	–0.63	0
OfSDG26	LYG015908	Chr8	nucleus	723	80.05	8.39	44.66	67.39	–0.57	0
OfSDG27	LYG016481	Chr8	endomembrane system	1052	119.97	6.37	47.27	82.02	–0.40	1
OfSDG28	LYG018763	Chr10	nucleus	1094	122.69	7.90	47.17	71.09	–0.40	0
OfSDG29	LYG018814	Chr10	nucleus	564	63.30	4.87	45.43	90.07	–0.27	0
OfSDG30	LYG019949	Chr11	endomembrane system	486	55.26	6.82	49.14	92.26	–0.24	0
OfSDG31	LYG021310	Chr11	chloroplast	594	66.92	7.16	37.56	76.08	–0.34	0
OfSDG32	LYG021991	Chr12	extracellular space	258	29.54	5.57	62.69	78.26	–0.34	0
OfSDG33	LYG023160	Chr13	nucleus	759	84.78	5.42	51.30	72.54	–0.60	0
OfSDG34	LYG023217	Chr13	extracellular space	258	29.30	7.87	37.37	74.03	–0.40	0
OfSDG35	LYG023961	Chr13	organelle membrane	560	63.25	5.66	42.07	86.18	–0.31	0
OfSDG36	LYG024220	Chr13	nucleus	318	35.92	7.93	54.80	71.45	–0.58	0
OfSDG37	LYG024238	Chr13	chloroplast	1086	122.18	8.91	52.27	80.37	–0.37	0
OfSDG38	LYG025021	Chr14	nucleus	698	76.36	8.52	50.59	74.66	–0.38	0
OfSDG39	LYG025066	Chr14	nucleus	549	62.36	4.88	35.28	93.77	–0.20	0
OfSDG40	LYG025772	Chr14	nucleus	336	36.91	7.80	56.17	82.11	–0.25	0
OfSDG41	LYG025965	Chr14	nucleus	507	56.59	4.94	52.98	86.94	–0.22	0
OfSDG42	LYG026106	Chr14	chloroplast	559	62.66	6.63	48.24	88.23	–0.23	0
OfSDG43	LYG026216	Chr14	nucleus	1632	183.02	5.68	47.48	80.54	–0.45	0
OfSDG44	LYG026245	Chr14	nucleus	843	92.73	8.06	50.73	72.34	–0.58	0
OfSDG45	LYG027218	Chr15	nucleus	870	98.15	6.42	43.22	74.39	–0.48	0
OfSDG46	LYG027233	Chr15	nucleus	663	74.62	6.01	44.60	79.92	–0.31	0
OfSDG47	LYG027598	Chr15	nucleus	898	101.23	8.16	42.40	75.02	–0.46	0
OfSDG48	LYG027905	Chr15	nucleus	959	106.44	5.32	50.47	77.45	–0.50	0
OfSDG49	LYG030093	Chr17	endomembrane system	498	56.48	5.68	49.53	89.74	–0.23	0
OfSDG50	LYG030097	Chr17	endomembrane system	534	60.86	6.01	47.63	87.90	–0.27	0
OfSDG51	LYG030692	Chr17	nucleus	1203	134.96	6.92	50.06	76.36	–0.53	0
OfSDG52	LYG031493	Chr18	nucleus	514	58.79	8.07	53.01	70.70	–0.54	0
OfSDG53	LYG031628	Chr18	nucleus	711	78.33	7.98	48.37	70.07	–0.46	0
OfSDG54	LYG032384	Chr19	mitochondri	478	52.96	5.64	42.96	99.85	–0.08	0

			al membrane							
OfSDG55	LYG032626	Chr19	chloroplast inner membrane	495	56.39	4.89	40.55	98.67	–0.21	0
OfSDG56	LYG033301	Chr19	nucleus	647	71.52	8.43	36.35	70.93	–0.45	0
OfSDG57	LYG033894	Chr20	nucleus	1047	119.11	8.49	44.98	74.39	–0.59	0
OfSDG58	LYG034245	Chr20	nucleus	661	74.29	6.23	48.58	77.55	–0.33	0
OfSDG59	LYG034822	Chr20	nucleus	2087	229.48	8.47	45.76	71.44	–0.47	0
OfSDG60	LYG034863	Chr20	endomembrane system	682	77.39	5.67	42.16	80.04	–0.34	0
OfSDG61	LYG034960	Chr21	nucleus	1040	118.46	8.29	39.96	76.39	–0.49	0
OfSDG62	LYG037486	Chr23	nucleus	1489	166.20	8.13	48.64	64.49	–0.76	0
OfSDG63	LYG038215	Chr23	nucleus	1197	134.94	8.22	47.95	72.06	–0.39	0
OfSDG64	LYG038562	Chr23	nucleus	638	71.19	6.20	51.05	72.02	–0.60	0
OfSDG65	LYG038643	unanchor5	nucleus	1106	125.61	8.36	44.66	74.21	–0.58	0
OfSDG66	LYG039561	unanchor13	nucleus	711	78.33	7.98	48.80	70.76	–0.45	0
OfSDG67	LYG040095	unanchor83	nucleus	408	46.21	8.54	57.76	75.49	–0.47	0
OfSDG68	LYG040179	unanchor81	nucleus	288	32.57	9.16	33.75	83.58	–0.27	0
OfSDG69	LYG040620	unanchor50	nucleus	1181	133.14	8.39	49.35	69.74	–0.43	0
OfHDMA1	LYG000906	Chr1	endomembrane system	2136	233.73	5.62	43.52	79.64	–0.43	0
OfHDMA2	LYG001626	Chr1	nucleus	573	64.29	5.37	37.28	69.79	–0.66	0
OfHDMA3	LYG002081	Chr1	nucleus	779	85.73	5.79	39.76	82.63	–0.41	0
OfHDMA4	LYG005137	Chr2	nucleus	776	84.97	5.98	44.23	72.80	–0.48	0
OfHDMA5	LYG007478	Chr4	nucleus	733	81.33	6.73	43.22	90.18	–0.24	0
OfHDMA6	LYG008221	Chr4	nucleus	904	98.52	8.37	41.00	91.25	–0.14	0
OfHDMA7	LYG011504	Chr6	nucleus	760	84.38	6.98	42.52	89.14	–0.26	0
OfHDMA8	LYG011969	Chr6	nucleus	782	85.92	5.98	34.61	87.76	–0.19	0
OfHDMA9	LYG012007	Chr6	nucleus	1238	133.26	6.72	40.21	83.33	–0.31	0
OfHDMA10	LYG016882	Chr9	nucleus	771	85.04	5.94	47.96	90.77	–0.25	0
OfHDMA11	LYG017056	Chr9	nucleus	962	105.29	4.94	53.69	66.46	–0.71	0
OfHDMA12	LYG022349	Chr12	plasma membrane	1852	201.88	5.39	41.05	79.90	–0.42	0
OfHDMA13	LYG023005	Chr12	chloroplast	443	49.44	5.45	34.68	79.71	–0.43	0
OfHDMA14	LYG026895	Chr15	nucleus	500	54.90	5.07	49.05	71.46	–0.49	0
OfHDMA15	LYG028130	Chr16	nucleus	439	48.37	5.06	47.59	71.87	–0.50	0
OfHDMA16	LYG034833	Chr20	nucleus	477	51.96	5.27	41.85	70.00	–0.44	0
OfHDMA17	LYG037480	Chr23	nucleus	775	84.69	5.95	41.82	75.17	–0.48	0
OfJMJ1	LYG000472	Chr1	nucleus	1157	132.40	5.35	49.42	66.04	–0.98	0
OfJMJ2	LYG002921	Chr1	endomembr	1015	114.0	8.25	49.99	81.46	–0.43	0

			ane system		9					
OfJMJ3	LYG003005	Chr1	nucleus	514	59.25	6.03	57.83	68.27	–0.63	0
OfJMJ4	LYG003697	Chr2	nucleus	903	103.12	7.74	55.94	74.82	–0.57	0
OfJMJ5	LYG004607	Chr2	plasma membrane	932	105.06	6.83	52.06	77.64	–0.42	0
OfJMJ6	LYG004726	Chr2	nucleus	1854	211.29	6.90	47.94	89.80	–0.29	0
OfJMJ7	LYG005626	Chr3	chloroplast	515	58.97	6.81	42.69	79.20	–0.42	0
OfJMJ8	LYG007295	Chr3	nucleus	1313	146.97	7.73	42.10	74.58	–0.62	0
OfJMJ9	LYG007660	Chr4	nucleus	1035	117.94	7.44	44.69	66.32	–0.91	0
OfJMJ10	LYG007763	Chr4	nucleus	954	108.23	5.92	51.60	75.67	–0.59	0
OfJMJ11	LYG008114	Chr4	nucleus	1231	138.76	7.64	52.20	73.44	–0.55	0
OfJMJ12	LYG008234	Chr4	nucleus	1412	157.97	6.63	48.46	74.00	–0.59	0
OfJMJ13	LYG008741	Chr4	endomembrane system	390	43.82	9.16	40.08	85.46	–0.51	0
OfJMJ14	LYG009683	Chr5	nucleus	1134	125.80	6.94	50.49	68.98	–0.60	0
OfJMJ15	LYG009854	Chr5	nucleus	1223	137.36	6.06	52.20	71.73	–0.56	0
OfJMJ16	LYG011985	Chr6	nucleus	1410	157.57	6.80	48.26	72.80	–0.58	0
OfJMJ17	LYG012023	Chr6	nucleus	1413	157.74	6.94	48.29	72.09	–0.59	0
OfJMJ18	LYG013152	Chr6	chloroplast	514	58.96	7.93	54.26	74.38	–0.56	0
OfJMJ19	LYG015735	Chr8	nucleus	465	54.11	4.89	31.99	76.47	–0.30	0
OfJMJ20	LYG016353	Chr8	nucleus	1059	119.76	8.43	50.52	83.76	–0.41	0
OfJMJ21	LYG016462	Chr8	nucleus	1066	120.37	5.84	52.19	77.05	–0.42	0
OfJMJ22	LYG018515	Chr10	nucleus	678	77.26	7.67	52.44	77.08	–0.40	0
OfJMJ23	LYG020559	Chr11	nucleus	135	14.79	8.93	50.49	65.85	–0.58	0
OfJMJ24	LYG020561	Chr11	nucleus	135	14.79	8.93	50.49	65.85	–0.58	0
OfJMJ25	LYG021484	Chr11	endomembrane system	231	27.02	9.17	48.00	83.55	–0.10	0
OfJMJ26	LYG024600	Chr13	nucleus	1411	157.57	9.08	51.31	66.67	–0.65	0
OfJMJ27	LYG030037	Chr17	nucleus	1122	127.69	5.81	47.29	87.80	–0.35	0
OfJMJ28	LYG032892	Chr19	nucleus	808	91.20	6.30	46.22	75.56	–0.39	0
OfJMJ29	LYG033290	Chr19	endomembrane system	317	35.62	8.72	42.85	91.32	–0.23	0
OfJMJ30	LYG033293	Chr19	nucleus	228	25.25	8.69	39.13	79.61	–0.54	0
OfJMJ31	LYG033295	Chr19	nucleus	215	24.29	9.35	54.57	83.86	–0.40	0
OfJMJ32	LYG035533	Chr21	endomembrane system	262	29.99	7.73	45.27	94.89	–0.22	0
OfJMJ33	LYG037577	Chr23	nucleus	135	14.80	9.43	48.50	65.85	–0.59	0
OfJMJ34	LYG039632	unanchor20	nucleus	861	96.85	6.81	49.29	75.55	–0.44	0

OfHAG1	LYG000359	Chr1	chloroplast	601	66.25	6.99	38.04	90.42	–0.13	0
OfHAG2	LYG002065	Chr1	nucleus	984	110.28	8.50	46.36	74.75	–0.68	0
OfHAG3	LYG002261	Chr1	chloroplast	273	30.32	9.34	41.00	78.94	–0.16	0
OfHAG4	LYG002391	Chr1	nucleus	563	63.14	8.71	32.18	85.52	–0.33	0
OfHAG5	LYG005126	Chr2	nucleus	415	45.69	4.46	53.57	89.52	–0.12	0
OfHAG6	LYG005882	Chr3	nucleus	283	32.61	9.15	36.34	65.12	–0.59	0
OfHAG7	LYG006252	Chr3	chloroplast	174	20.34	6.91	43.60	82.36	–0.37	0
OfHAG8	LYG008233	Chr4	cytoplasm	210	23.12	5.63	56.16	96.67	–0.01	0
OfHAG9	LYG009066	Chr4	nucleus	460	51.74	5.61	42.48	98.02	–0.26	0
OfHAG10	LYG010659	Chr5	chloroplast	249	27.92	5.23	46.51	93.17	–0.23	0
OfHAG11	LYG011984	Chr6	chloroplast	295	33.13	6.67	55.36	87.25	–0.09	0
OfHAG12	LYG012033	Chr6	nucleus	305	34.12	9.56	31.73	84.72	–0.25	0
OfHAG13	LYG014084	Chr7	nucleus	157	17.98	5.96	47.91	87.39	–0.29	0
OfHAG14	LYG015110	Chr8	nucleus	400	45.32	8.84	40.68	88.65	–0.24	0
OfHAG15	LYG015754	Chr8	nucleus	563	63.17	8.72	36.59	85.52	–0.32	0
OfHAG16	LYG015856	Chr8	chloroplast	330	36.69	7.09	49.92	87.18	–0.34	0
OfHAG17	LYG018573	Chr10	nucleus	263	30.37	9.33	51.29	77.15	–0.50	0
OfHAG18	LYG019695	Chr10	nucleus	157	18.17	9.41	40.08	82.04	–0.53	0
OfHAG19	LYG020611	Chr11	chloroplast	608	67.20	8.45	38.46	95.41	–0.14	0
OfHAG20	LYG020624	Chr11	cytoplasm	404	45.32	9.08	33.66	89.95	–0.10	0
OfHAG21	LYG020917	Chr11	cytoplasm	192	22.00	6.97	28.70	86.77	–0.17	0
OfHAG22	LYG021822	Chr12	nucleus	418	46.66	9.36	35.70	88.54	–0.17	0
OfHAG23	LYG023941	Chr13	cytoplasm	466	52.33	6.20	37.04	91.61	–0.15	0
OfHAG24	LYG024175	Chr13	chloroplast	253	29.11	8.70	52.89	98.18	–0.07	0
OfHAG25	LYG024973	Chr14	nucleus	655	72.62	8.34	40.13	83.36	–0.33	0
OfHAG26	LYG025940	Chr14	nucleus	268	31.08	7.68	40.99	91.27	–0.38	0
OfHAG27	LYG026762	Chr15	nucleus	460	51.86	5.96	37.98	96.54	–0.24	0
OfHAG28	LYG028991	Chr16	nucleus	186	21.15	6.52	39.39	74.57	–0.49	0
OfHAG29	LYG032709	Chr19	chloroplast	312	35.80	8.94	60.12	77.18	–0.52	0
OfHAG30	LYG034607	Chr20	chloroplast	165	18.46	8.79	26.50	93.39	–0.10	0
OfHAG31	LYG035162	Chr21	chloroplast	105	11.73	10.41	40.48	85.52	–0.19	0
OfHAG32	LYG036049	Chr21	chloroplast	206	23.12	10.03	30.04	91.31	–0.26	0
OfHAG33	LYG037470	Chr23	nucleus	845	93.99	8.82	39.59	75.49	–0.63	0
OfHAG34	LYG038035	Chr23	nucleus	187	21.34	6.56	40.85	74.65	–0.50	0
OfHAG35	LYG038364	Chr23	chloroplast	302	35.10	9.68	53.93	79.11	–0.46	0
OfHAG36	LYG038448	Chr23	chloroplast	474	53.06	5.94	41.71	74.47	–0.54	0
OfHAG37	LYG039030	unanchor43	chloroplast	174	20.34	6.91	42.50	82.36	–0.37	0
OfHAG38	LYG039460	unanchor72	nucleus	157	18.17	9.41	40.08	82.04	–0.53	0
OfHAG39	LYG041063	unanchor22	nucleus	400	45.37	8.73	41.84	86.70	–0.27	0
OfHAM1	LYG002043	Chr1	nucleus	441	51.04	6.92	38.70	81.09	–0.55	0
OfHAM2	LYG015395	Chr8	nucleus	442	51.23	7.17	42.12	80.68	–0.56	0
OfHAC1	LYG002157	Chr1	nucleus	1514	168.89	7.56	50.84	66.10	–0.68	0
OfHAC2	LYG008065	Chr4	nucleus	1597	179.96	8.54	53.00	66.71	–0.69	0
OfHAC3	LYG012446	Chr6	nucleus	1585	178.86	7.93	53.48	63.61	–0.76	0

OfHAC4	LYG015505	Chr8	nucleus	1726	193.05	7.41	50.09	65.54	–0.68	0
OfHAC5	LYG020645	Chr11	nucleus	1343	151.61	6.34	48.35	75.91	–0.40	0
OfHAC6	LYG031907	Chr18	nucleus	366	41.30	5.58	46.86	73.80	–0.46	0
OfHAC7	LYG037335	Chr22	nucleus	1740	195.65	7.72	54.84	71.34	–0.60	0
OfHAF1	LYG029210	Chr16	nucleus	1869	212.34	5.68	52.60	73.12	–0.83	0
OfHDA1	LYG000507	Chr1	plasma membrane	488	55.45	5.89	36.91	79.10	–0.29	0
OfHDA2	LYG002367	Chr1	plasma membrane	435	49.18	5.42	44.93	77.26	–0.41	0
OfHDA3	LYG006400	Chr3	endomembrane system	448	50.98	5.21	37.34	91.14	–0.20	0
OfHDA4	LYG006432	Chr3	organelle membrane	392	42.97	5.37	28.94	82.83	–0.21	0
OfHDA5	LYG009590	Chr5	plasma membrane	659	73.41	5.61	46.33	83.75	–0.28	0
OfHDA6	LYG009594	Chr5	endomembrane system	567	62.20	5.14	41.24	79.72	–0.10	0
OfHDA7	LYG015736	Chr8	nucleus	98	11.47	10.55	85.26	74.69	–0.53	0
OfHDA8	LYG021965	Chr12	plasma membrane	507	57.38	5.00	41.56	74.18	–0.53	0
OfHDA9	LYG021972	Chr12	organelle membrane	438	48.95	7.64	35.95	80.39	–0.27	0
OfHDA10	LYG024674	Chr13	nucleus	593	64.92	5.36	43.35	80.19	–0.19	1
OfHDA11	LYG025983	Chr14	plasma membrane	511	57.62	5.00	41.16	69.77	–0.59	0
OfHDA12	LYG026597	Chr15	organelle membrane	360	39.75	7.05	38.02	97.25	–0.08	0
OfHDA13	LYG026884	Chr15	plasma membrane	466	52.65	5.27	37.81	73.84	–0.54	0
OfHDA14	LYG034454	Chr20	nucleus	474	51.52	8.63	30.12	69.30	–0.34	0
OfHDA15	LYG034828	Chr20	plasma membrane	441	50.05	5.12	40.39	73.13	–0.56	0
OfHDA16	LYG035046	Chr21	endomembrane system	430	49.04	5.02	34.21	82.95	–0.38	0
OfHDA17	LYG039236	unanchor23	endomembrane system	436	49.79	5.10	34.66	82.71	–0.42	0
OfHDT1	LYG006949	Chr3	nucleus	301	32.92	4.97	42.66	51.26	–1.11	0
OfHDT2	LYG006950	Chr3	nucleus	303	32.98	4.93	47.25	48.32	–1.14	0
OfHDT3	LYG010481	Chr5	nucleus	200	22.05	4.33	45.90	51.25	–1.14	0
OfHDT4	LYG023316	Chr13	nucleus	163	18.10	4.11	46.49	62.82	–0.76	0
OfHDT5	LYG028009	Chr15	nucleus	128	14.74	5.69	24.71	100.47	–0.08	0
OfHDT6	LYG034455	Chr20	nucleus	100	12.01	4.43	20.21	77.00	–0.07	0
OfHDT7	LYG035725	Chr21	nucleus	305	33.35	4.88	47.17	48.62	–1.13	0
OfSRT1	LYG000493	Chr1	chloroplast	395	44.35	9.09	41.44	78.05	–0.33	0
OfSRT2	LYG008087	Chr4	nucleus	473	53.17	9.29	43.65	88.77	–0.19	0
OfSRT3	LYG016225	Chr8	nucleus	508	56.42	9.13	48.59	87.80	–0.24	0

[1] molecular weights; [2] isoelectric points; [3] instability indexe; [4] grand average of hydropathicity; [5] protein transmembrane domains.

Table S2. The Ka/Ks values of segmental and tandem duplications between *OfHMs*.

Type	Number	Gene 1	Gene 2	Ks	Ka	Ka/Ks
tandem duplications	1	*OfHDT2*	*OfHDT1*	0.975	0.042	0.043
	2	*OfSDG49*	*OfSDG50*	1.648	0.961	0.583
segmental duplications	3	*OfJMJ23*	*OfJMJ24*	0	0	#DIV/0!
	4	*OfHDA1*	*OfHDA8*	0.177	0.044	0.245
	5	*OfHDMA1*	*OfHDMA12*	2.125	3.218	1.514
	6	*OfHDMA2*	*OfHDMA13*	n.a.	n.a.	#VALUE!
	7	*OfSDG3*	*OfSDG38*	0.403	0.044	0.108
	8	*OfSDG3*	*OfSDG53*	0.360	0.198	0.550
	9	*OfHAG2*	*OfHAG5*	1.484	n.a.	#VALUE!
	10	*OfHDMA3*	*OfHDMA4*	0.498	0.134	0.269
	11	*OfSDG1*	*OfSDG6*	n.a.	n.a.	#VALUE!
	12	*OfHAC1*	*OfHAC7*	0.945	1.977	2.091
	13	*OfHAG2*	*OfHAG33*	n.a.	2.068	#VALUE!
	14	*OfHDMA3*	*OfHDMA17*	2.125	0.087	0.041
	15	*OfSDG1*	*OfSDG62*	n.a.	n.a.	#VALUE!
	16	*OfSDG3*	*OfSDG62*	0.371	0.142	0.383
	17	*OfHAC1*	*OfHAC2*	1.207	1.431	1.185
	18	*OfHAC1*	*OfHAC3*	0.585	n.a.	#VALUE!
	19	*OfHAM1*	*OfHAM2*	0.189	0.000	0.000
	20	*OfHAC1*	*OfHAC4*	n.a.	1.618	#VALUE!
	21	*OfHDA2*	*OfHDA7*	n.a.	1.491	#VALUE!
	22	*OfHAG4*	*OfHAG15*	0.390	0.000	0.000
	23	*OfSDG3*	*OfSDG26*	0.425	0.089	0.210
	24	*OfJMJ2*	*OfJMJ20*	n.a.	1.403	#VALUE!
	25	*OfJMJ3*	*OfJMJ21*	n.a.	1.341	#VALUE!
	26	*OfHAG17*	*OfHAG35*	1.576	2.232	1.416
	27	*OfSDG28*	*OfSDG63*	0.360	0.045	0.125
	28	*OfSDG31*	*OfSDG46*	1.727	1.107	0.641
	29	*OfSDG31*	*OfSDG58*	n.a.	1.116	#VALUE!
	30	*OfSDG33*	*OfSDG48*	n.a.	1.309	#VALUE!
	31	*OfSDG37*	*OfSDG10*	n.a.	1.543	#VALUE!
	32	*OfSDG33*	*OfSDG18*	n.a.	0.775	#VALUE!
	33	*OfHDT4*	*OfHDT3*	0.417	0.089	0.214
	34	*OfSDG36*	*OfSDG17*	0.511	2.192	4.293
	35	*OfJMJ26*	*OfJMJ14*	0.327	0.397	1.216
	36	*OfSDG38*	*OfSDG53*	1.171	0.142	0.121
	37	*OfSDG38*	*OfSDG7*	1.246	0.091	0.073
	38	*OfSDG41*	*OfSDG23*	2.622	0.922	0.352
	39	*OfSDG43*	*OfSDG24*	0.000	0.084	#DIV/0!
	40	*OfSDG38*	*OfSDG26*	0.845	0.043	0.051
	41	*OfPRMT3*	*OfPRMT11*	0.586	0.349	0.595
	42	*OfHDA13*	*OfHDA15*	0.425	0.000	0.000
	43	*OfHDMA14*	*OfHDMA16*	0.000	0.087	#DIV/0!
	44	*OfSDG46*	*OfSDG58*	0.149	0.000	0.000
	45	*OfPRMT5*	*OfPRMT10*	0.167	0.044	0.263
	46	*OfSDG47*	*OfSDG57*	0.469	0.291	0.619
	47	*OfSDG45*	*OfSDG18*	0.383	0.868	2.266
	48	*OfSDG48*	*OfSDG18*	0.666	0.160	0.240

49	*OfHAG28*	*OfHAG34*	n.a.	0.000	#VALUE!
50	*OfHDMA15*	*OfHDMA11*	n.a.	0.778	#VALUE!
51	*OfPRMT6*	*OfPRMT2*	0.449	0.243	0.541
52	*OfSDG53*	*OfSDG7*	0.000	0.045	#DIV/0!
53	*OfSDG53*	*OfSDG26*	0.396	0.193	0.488
54	*OfJMJ28*	*OfJMJ5*	0.000	0.046	#DIV/0!
55	*OfPRMT9*	*OfPRMT1*	0.717	0.175	0.244
56	*OfSDG56*	*OfSDG4*	0.776	0.715	0.921
57	*OfHAG5*	*OfHAG33*	0.433	n.a.	#VALUE!
58	*OfHDMA4*	*OfHDMA17*	0.458	0.000	0.000
59	*OfSDG6*	*OfSDG62*	0.428	0.235	0.548
60	*OfHDA16*	*OfHDA3*	1.648	0.000	0.000
61	*OfHDT7*	*OfHDT1*	0.224	0.085	0.381
62	*OfJMJ8*	*OfJMJ18*	1.570	2.214	1.410
63	*OfSDG7*	*OfSDG26*	0.410	0.139	0.339
64	*OfHDMA6*	*OfHDMA9*	0.766	0.224	0.292
65	*OfJMJ12*	*OfJMJ17*	0.163	0.000	0.000
66	*OfHDMA5*	*OfHDMA7*	1.648	0.596	0.362
67	*OfHAG8*	*OfHAG11*	0.572	n.a.	#VALUE!
68	*OfJMJ12*	*OfJMJ16*	0.163	0.000	0.000
69	*OfSDG12*	*OfSDG21*	0.224	0.132	0.588
70	*OfHAC2*	*OfHAC3*	1.734	n.a.	#VALUE!
71	*OfJMJ10*	*OfJMJ20*	n.a.	n.a.	#VALUE!
72	*OfHAC2*	*OfHAC4*	0.907	1.313	1.447
73	*OfHAC3*	*OfHAC4*	1.327	1.420	1.070
74	*OfSDG3*	*OfSDG66*	0.360	0.198	0.550
75	*OfHAG18*	*OfHAG37*	0.000	0.000	#DIV/0!
76	*OfSDG28*	*OfSDG67*	0.360	0.045	0.125
77	*OfSDG32*	*OfSDG69*	1.060	0.664	0.626
78	*OfSDG47*	*OfSDG65*	1.309	n.a.	#VALUE!
79	*OfSDG53*	*OfSDG66*	0.000	0.000	#DIV/0!
80	*OfJMJ28*	*OfJMJ34*	0.000	0.046	#DIV/0!
81	*OfJMJ5*	*OfJMJ34*	0.000	0.000	#DIV/0!
82	*OfSDG57*	*OfSDG65*	0.937	2.162	2.308
83	*OfHDA16*	*OfHDA17*	1.648	0.000	0.000
84	*OfSDG63*	*OfSDG67*	0.000	0.000	#DIV/0!
85	*OfSDG7*	*OfSDG66*	0.000	0.045	#DIV/0!
86	*OfHAG7*	*OfHAG39*	0.131	0.048	0.364
87	*OfHDA3*	*OfHDA17*	0.000	0.000	#DIV/0!
88	*OfSDG26*	*OfSDG66*	0.396	0.193	0.488
89	*OfHAG14*	*OfHAG38*	0.000	0.000	#DIV/0!

Table S3. The Ka/Ks values of segmental duplications between *OfHMs* and *AtHMs*.

Number	Gene 1	Gene 2	Ks	Ka	Ka/Ks
1	*AtPRMT16*	*OfPRMT4*	2.153	n.a.	#VALUE!
2	*AtPRMT13*	*OfPRMT5*	1.435	1.341	0.934
3	*AtPRMT14*	*OfPRMT5*	n.a.	1.902	#VALUE!
4	*AtPRMT12*	*OfPRMT7*	2.089	n.a.	#VALUE!
5	*AtPRMT13*	*OfPRMT10*	1.900	1.594	0.839
6	*AtPRMT14*	*OfPRMT10*	n.a.	1.895	#VALUE!
7	*AtSDG8*	*OfSDG1*	n.a.	2.009	#VALUE!
8	*AtSDG8*	*OfSDG6*	1.415	1.727	1.220
9	*AtSDG30*	*OfSDG9*	1.061	n.a.	#VALUE!
10	*AtSDG27*	*OfSDG9*	1.016	3.272	3.220
11	*AtSDG16*	*OfSDG10*	n.a.	1.870	#VALUE!
12	*AtSDG31*	*OfSDG14*	n.a.	2.355	#VALUE!
13	*AtSDG34*	*OfSDG17*	1.263	3.013	2.386
14	*AtSDG13*	*OfSDG18*	2.125	2.125	1.000
15	*AtSDG3*	*OfSDG18*	1.584	2.391	1.510
16	*AtSDG31*	*OfSDG18*	1.672	n.a.	#VALUE!
17	*AtSDG18*	*OfSDG18*	n.a.	n.a.	#VALUE!
18	*AtSDG38*	*OfSDG22*	1.712	3.400	1.986
19	*AtSDG6*	*OfSDG24*	3.349	2.460	0.734
20	*AtSDG14*	*OfSDG28*	0.646	2.806	4.348
21	*AtSDG3*	*OfSDG31*	n.a.	1.431	#VALUE!
22	*AtSDG15*	*OfSDG32*	n.a.	2.283	#VALUE!
23	*AtSDG18*	*OfSDG33*	n.a.	n.a.	#VALUE!
24	*AtSDG35*	*OfSDG35*	1.095	1.648	1.505
25	*AtSDG34*	*OfSDG36*	n.a.	1.423	#VALUE!
26	*AtSDG14*	*OfSDG37*	n.a.	n.a.	#VALUE!
27	*AtSDG16*	*OfSDG37*	n.a.	1.215	#VALUE!
28	*AtSDG29*	*OfSDG37*	1.092	1.882	1.724
29	*AtSDG6*	*OfSDG43*	3.349	2.460	0.734
30	*AtSDG1*	*OfSDG44*	n.a.	1.772	#VALUE!
31	*AtSDG13*	*OfSDG45*	n.a.	1.936	#VALUE!
32	*AtSDG18*	*OfSDG45*	n.a.	n.a.	#VALUE!
33	*AtSDG3*	*OfSDG46*	n.a.	1.338	#VALUE!
34	*AtSDG22*	*OfSDG46*	n.a.	1.148	#VALUE!
35	*AtSDG13*	*OfSDG48*	2.622	2.031	0.774
36	*AtSDG22*	*OfSDG48*	n.a.	1.807	#VALUE!
37	*AtSDG4*	*OfSDG52*	0.781	n.a.	#VALUE!
38	*AtSDG43*	*OfSDG55*	0.628	n.a.	#VALUE!
39	*AtSDG30*	*OfSDG57*	1.623	n.a.	#VALUE!
40	*AtSDG27*	*OfSDG57*	n.a.	n.a.	#VALUE!
41	*AtSDG3*	*OfSDG58*	n.a.	1.256	#VALUE!
42	*AtSDG22*	*OfSDG58*	n.a.	1.093	#VALUE!

43	*AtSDG30*	*OfSDG61*	n.a.	n.a.	#VALUE!
44	*AtSDG27*	*OfSDG61*	n.a.	n.a.	#VALUE!
45	*AtSDG8*	*OfSDG62*	1.605	1.170	0.729
46	*AtSDG5*	*OfSDG64*	2.814	0.609	0.216
47	*AtSDG10*	*OfSDG64*	1.263	2.142	1.696
48	*AtHDMA1*	*OfHDMA6*	n.a.	2.322	#VALUE!
49	*AtHDMA1*	*OfHDMA9*	1.545	1.680	1.087
50	*AtJMJ28*	*OfJMJ2*	0.743	0.740	0.996
51	*AtJMJ18*	*OfJMJ3*	1.798	1.611	0.896
52	*AtJMJ15*	*OfJMJ3*	n.a.	3.212	#VALUE!
53	*AtJMJ29*	*OfJMJ4*	1.052	2.515	2.391
54	*AtJMJ26*	*OfJMJ4*	0.890	1.587	1.782
55	*AtJMJ17*	*OfJMJ6*	2.472	1.729	0.700
56	*AtJMJ11*	*OfJMJ12*	2.716	1.197	0.441
57	*AtJMJ12*	*OfJMJ14*	1.309	n.a.	#VALUE!
58	*AtJMJ11*	*OfJMJ16*	1.291	1.380	1.068
59	*AtJMJ11*	*OfJMJ17*	1.291	1.380	1.068
60	*AtJMJ20*	*OfJMJ19*	0.884	1.985	2.246
61	*AtJMJ28*	*OfJMJ20*	0.724	0.897	1.240
62	*AtJMJ18*	*OfJMJ21*	n.a.	n.a.	#VALUE!
63	*AtJMJ15*	*OfJMJ21*	1.493	n.a.	#VALUE!
64	*AtJMJ12*	*OfJMJ26*	n.a.	1.526	#VALUE!
65	*AtJMJ13*	*OfJMJ28*	1.648	2.321	1.409
66	*AtHAG3*	*OfHAG4*	1.463	2.022	1.382
67	*AtHAG2*	*OfHAG9*	n.a.	n.a.	#VALUE!
68	*AtHAG3*	*OfHAG15*	1.838	1.853	1.008
69	*AtHDA15*	*OfHDA1*	1.424	n.a.	#VALUE!
70	*AtHDA6*	*OfHDA2*	n.a.	2.237	#VALUE!
71	*AtHDA10*	*OfHDA3*	1.091	n.a.	#VALUE!
72	*AtHDA8*	*OfHDA4*	n.a.	2.277	#VALUE!
73	*AtHDA6*	*OfHDA7*	n.a.	2.616	#VALUE!
74	*AtHDA15*	*OfHDA9*	n.a.	1.899	#VALUE!
75	*AtHDA2*	*OfHDA12*	1.549	1.970	1.272
76	*AtHDA10*	*OfHDA16*	1.263	n.a.	#VALUE!
77	*AtHDT3*	*OfHDT1*	0.949	n.a.	#VALUE!
78	*AtHDT1*	*OfHDT3*	n.a.	n.a.	#VALUE!
79	*AtHDT2*	*OfHDT3*	1.952	n.a.	#VALUE!
80	*AtHDT1*	*OfHDT4*	n.a.	2.290	#VALUE!
81	*AtHDT2*	*OfHDT4*	n.a.	n.a.	#VALUE!
82	*AtHDT3*	*OfHDT7*	1.392	n.a.	#VALUE!
83	*AtSRT1*	*OfSRT3*	2.429	n.a.	#VALUE!
84	*AtSDG30*	*OfSDG65*	1.623	n.a.	#VALUE!
85	*AtSDG27*	*OfSDG65*	n.a.	n.a.	#VALUE!
86	*AtSDG15*	*OfSDG69*	n.a.	n.a.	#VALUE!
87	*AtHDA10*	*OfHDA17*	n.a.	3.226	#VALUE!

Table S4. Differentially expressed *OfHMs* detected were under Aza or ethylene treatment.

Gene	CK_1d	CK_5d	CK_8d	Aza_1d	Aza_5d	Aza_8d	ETH_1d	ETH_5d	ETH_8d
OfPRMT1	9.90	11.72	12.85	18.71	14.56	11.37	16.86	14.91	15.99
OfPRMT2	0.60	0.09	0.27	0.42	0.27	1.35	0.69	0.21	0.36
OfPRMT4	1.77	0.52	0.80	2.82	1.22	0.39	1.88	0.70	0.72
OfPRMT5	20.28	14.99	15.33	18.94	15.84	8.88	19.31	12.86	12.06
OfPRMT6	13.09	2.30	1.35	4.10	1.30	2.87	3.03	0.47	0.68
OfPRMT7	5.98	2.25	3.43	7.25	3.76	4.53	6.51	2.90	1.99
OfPRMT8	35.51	18.08	18.41	40.76	25.78	26.00	36.38	25.16	22.88
OfPRMT9	1.81	0.79	0.47	1.17	0.72	1.17	0.82	0.46	0.66
OfPRMT10	4.84	2.29	2.74	4.42	2.06	1.71	4.35	1.42	1.99
OfPRMT12	6.65	1.48	3.69	10.36	3.95	3.27	8.59	2.88	3.27
OfSDG2	1.90	0.66	1.56	6.87	2.87	0.93	7.71	2.46	0.81
OfSDG3	7.82	5.41	6.50	7.55	5.87	4.74	7.32	5.05	5.06
OfSDG4	4.79	1.97	2.98	5.11	2.41	1.73	4.19	2.11	2.15
OfSDG5	1.84	1.82	1.90	1.03	0.73	0.42	1.31	0.98	0.89
OfSDG7	8.84	4.34	6.12	13.52	6.74	3.97	12.81	7.83	5.56
OfSDG9	2.20	2.37	2.21	0.85	3.39	0.51	1.01	3.42	1.18
OfSDG10	4.54	2.96	4.23	2.58	2.00	4.17	2.55	2.04	2.37
OfSDG11	1.20	1.79	1.61	1.71	1.33	2.43	1.60	0.87	1.27
OfSDG12	3.41	7.52	5.37	1.29	5.21	2.02	1.57	12.31	4.60
OfSDG13	3.62	1.44	1.09	2.80	1.12	0.79	2.16	0.64	0.55
OfSDG15	0.67	0.00	0.15	1.22	0.53	0.03	0.81	0.31	0.17
OfSDG16	1.29	1.05	1.03	0.70	1.09	0.94	0.79	0.77	0.76
OfSDG18	1.37	0.34	0.95	1.30	0.85	0.71	1.19	0.24	0.36
OfSDG19	6.46	1.46	4.01	15.83	7.74	4.79	13.56	3.24	3.41
OfSDG20	6.69	0.81	3.07	13.58	7.12	3.31	11.72	3.56	3.21
OfSDG21	1.88	1.65	1.86	0.66	0.87	0.57	0.90	1.11	1.24
OfSDG22	5.73	3.06	6.14	13.43	13.71	4.59	12.04	10.95	8.07
OfSDG23	1.25	0.60	0.87	2.14	0.84	0.33	1.99	0.63	0.63
OfSDG24	1.60	1.37	0.81	0.70	0.51	0.59	0.49	0.42	0.49
OfSDG25	3.29	1.55	0.85	1.02	0.48	1.25	1.05	0.37	0.63
OfSDG26	7.40	3.90	3.66	5.60	4.05	1.92	5.33	3.04	2.43
OfSDG27	12.76	1.28	6.88	9.45	3.97	4.43	8.63	3.43	5.23
OfSDG28	1.54	1.51	1.20	0.53	0.65	0.74	0.83	0.58	0.48
OfSDG29	3.81	1.92	0.99	3.22	1.30	0.09	3.11	0.68	0.33
OfSDG33	2.86	1.08	1.69	2.24	1.14	1.31	1.80	1.12	1.02
OfSDG34	5.21	2.86	3.66	6.17	3.55	2.35	5.64	3.44	2.13
OfSDG38	2.11	0.40	0.24	1.86	0.26	0.13	1.49	0.11	0.05
OfSDG39	2.65	0.50	0.32	0.81	0.37	0.32	1.34	0.53	0.27
OfSDG40	4.64	3.65	5.59	5.34	5.95	5.57	6.63	6.73	6.92
OfSDG42	15.88	7.25	10.38	21.45	13.12	4.71	18.97	12.80	9.43
OfSDG43	1.09	1.68	0.78	0.62	0.90	0.62	0.52	0.51	0.29

OfSDG44	3.02	1.53	1.45	1.62	1.17	0.74	1.43	0.91	1.21
OfSDG46	1.91	2.18	2.93	5.23	4.04	2.05	3.91	3.40	3.39
OfSDG48	4.13	2.10	1.30	2.32	0.90	0.80	1.94	1.21	0.65
OfSDG49	1.10	1.33	0.79	0.93	0.82	0.70	0.91	0.73	0.70
OfSDG51	1.12	1.03	1.12	0.54	0.56	0.44	0.62	0.58	0.35
OfSDG54	9.23	5.77	10.85	22.53	16.72	8.80	19.94	17.17	14.04
OfSDG55	8.07	2.30	3.27	19.14	6.87	0.97	15.38	5.12	2.14
OfSDG56	1.66	1.43	1.49	0.97	1.06	1.65	1.33	0.90	1.34
OfSDG58	1.23	1.85	1.81	2.13	2.18	0.55	1.98	1.72	2.02
OfSDG60	2.15	1.51	1.50	1.08	1.85	0.79	1.28	1.45	1.14
OfSDG62	1.95	1.81	1.05	0.53	0.53	0.91	0.65	0.47	0.59
OfSDG64	2.40	4.68	5.62	0.90	2.56	6.05	1.02	3.26	6.23
OfHDMA2	5.93	5.81	4.30	2.81	2.43	3.26	2.49	2.25	2.13
OfHDMA3	3.60	3.41	3.49	2.83	3.15	1.99	3.01	4.08	3.00
OfHDMA4	8.67	5.55	6.90	13.81	7.47	7.46	12.56	7.03	5.56
OfHDMA10	1.65	0.68	1.71	4.87	2.76	2.21	4.22	2.38	2.17
OfHDMA11	5.47	4.09	4.05	1.91	1.89	1.13	1.69	2.10	2.06
OfHDMA14	15.33	14.59	14.96	17.33	18.60	18.00	16.68	19.07	17.76
OfHDMA15	5.14	3.05	2.11	1.36	1.75	1.64	1.54	1.25	1.67
OfHDMA16	1.82	3.29	5.20	2.99	4.05	2.25	3.54	5.25	4.08
OfHDMA17	6.54	5.16	5.00	7.70	6.49	3.65	7.69	5.71	3.08
OfJMJ1	2.00	0.69	0.67	0.65	0.17	0.06	0.79	0.38	0.31
OfJMJ7	1.85	0.65	0.87	2.12	0.65	1.15	1.79	0.86	0.63
OfJMJ10	2.17	2.07	1.20	0.70	0.38	0.47	0.75	0.46	0.40
OfJMJ11	3.59	2.82	1.60	0.99	0.64	0.73	1.15	0.73	0.64
OfJMJ15	4.05	3.86	3.13	1.52	1.62	1.29	1.79	1.35	1.45
OfJMJ19	5.12	2.62	2.40	4.86	3.42	1.72	4.52	2.84	1.80
OfJMJ20	0.40	10.50	2.20	0.18	0.51	3.14	0.18	0.59	1.27
OfJMJ22	2.60	2.75	2.42	1.62	2.27	0.96	1.40	1.54	1.66
OfJMJ26	1.42	0.72	0.42	0.38	0.17	0.63	0.51	0.20	0.25
OfJMJ27	18.23	19.70	34.23	23.17	33.45	22.94	22.24	30.70	24.55
OfJMJ28	5.83	5.65	2.70	2.19	1.39	0.60	1.68	1.39	1.24
OfJMJ34	1.27	1.88	1.43	1.06	0.68	0.35	1.09	0.85	0.75
OfHAG1	3.78	3.11	3.72	5.46	4.04	2.20	5.13	4.24	3.24
OfHAG2	2.74	1.93	1.95	1.61	0.78	1.41	1.38	0.97	0.88
OfHAG3	4.69	0.00	0.30	2.55	0.38	0.00	2.89	0.51	0.15
OfHAG4	9.97	10.58	21.75	15.10	20.42	22.20	17.14	23.77	33.93
OfHAG7	43.63	40.37	25.02	26.96	31.66	42.68	26.78	26.15	35.82
OfHAG8	37.49	55.58	127.77	54.59	118.08	79.01	60.62	107.06	121.71
OfHAG9	5.24	2.03	1.41	3.06	1.37	1.45	2.78	1.60	1.06
OfHAG10	14.35	12.26	11.75	13.52	15.51	11.91	16.60	16.45	14.64
OfHAG11	35.48	25.00	57.20	80.82	75.89	41.27	70.90	59.06	46.23
OfHAG12	18.53	14.19	14.11	16.73	18.32	14.94	20.70	21.66	17.22
OfHAG13	5.09	10.28	3.42	1.68	1.76	3.24	2.70	2.26	1.63
OfHAG15	5.40	5.37	9.64	9.94	10.62	10.29	10.20	9.65	12.37

OfHAG16	46.27	45.57	52.89	37.45	59.67	83.51	44.77	71.74	85.55
OfHAG17	1.58	4.14	2.84	1.16	1.42	2.76	1.25	2.35	0.99
OfHAG18	1.45	0.41	0.13	0.51	0.23	0.08	0.60	0.09	0.34
OfHAG19	4.38	3.30	2.81	5.43	3.54	2.09	5.61	3.45	2.29
OfHAG21	51.55	36.67	31.94	45.10	43.08	56.68	40.53	35.00	39.68
OfHAG22	0.15	1.43	1.84	0.21	0.87	0.89	0.00	0.71	1.10
OfHAG24	6.29	12.38	14.79	7.93	17.05	16.93	8.05	17.35	17.04
OfHAG26	1.69	1.34	1.66	1.00	0.37	0.48	0.95	0.72	0.57
OfHAG27	5.26	6.26	5.09	3.49	4.52	7.56	2.91	3.79	3.95
OfHAG28	35.59	19.76	24.95	36.41	25.12	31.23	33.09	25.38	23.05
OfHAG29	11.84	6.96	9.53	21.31	11.33	8.44	19.07	9.06	7.22
OfHAG30	24.33	10.34	8.70	22.14	14.27	10.09	18.82	10.57	9.12
OfHAG32	6.29	5.95	12.51	8.25	9.29	14.72	8.77	10.05	12.16
OfHAG34	57.39	44.68	59.65	55.28	58.88	90.87	57.37	55.08	53.65
OfHAG35	1.48	2.12	1.93	1.01	0.64	2.33	0.97	1.10	0.56
OfHAG37	2.83	0.42	0.91	1.07	0.66	0.47	0.87	0.84	0.69
OfHAG39	46.93	38.85	33.39	32.98	41.42	46.64	39.56	36.88	39.41
OfHAM1	24.40	33.94	35.94	24.03	30.04	26.95	22.34	35.01	32.57
OfHAM2	13.08	15.99	17.27	11.68	17.71	11.96	12.21	20.35	16.78
OfHAC1	3.18	2.68	3.13	1.07	1.13	1.15	1.12	1.32	1.41
OfHAC2	1.49	1.06	1.12	0.80	0.68	0.25	0.75	0.77	0.55
OfHAC3	3.81	2.63	2.48	1.15	1.13	0.71	1.42	1.17	1.45
OfHAC4	1.49	1.37	1.30	0.65	0.60	0.58	0.68	0.55	0.71
OfHAC6	1.94	1.96	1.36	0.57	0.31	2.78	0.32	0.80	0.98
OfHAC7	1.64	1.90	1.20	0.45	0.70	0.78	0.63	0.52	0.81
OfHAF1	1.14	1.08	1.38	0.93	1.79	0.55	0.98	1.33	0.75
OfHDA2	40.66	58.01	87.27	63.06	112.12	55.98	70.68	120.89	105.76
OfHDA3	21.33	16.35	9.85	12.98	12.43	8.80	15.82	11.77	12.42
OfHDA4	33.52	35.68	46.56	58.53	82.85	37.92	61.74	83.55	69.44
OfHDA5	7.14	9.37	8.50	4.53	4.34	2.36	5.43	4.91	4.67
OfHDA6	2.65	2.41	2.69	5.47	2.98	1.59	5.12	2.77	3.12
OfHDA9	13.00	13.18	16.51	18.62	21.22	11.85	20.88	22.90	19.07
OfHDA10	1.85	0.36	0.69	1.30	0.29	0.73	1.01	0.13	0.42
OfHDA12	3.70	7.47	6.18	6.01	8.09	8.27	6.78	8.80	7.72
OfHDA13	55.02	56.61	65.20	71.87	79.34	55.82	77.14	87.90	78.24
OfHDA15	67.12	70.93	91.96	90.50	101.30	107.26	87.63	114.97	124.93
OfHDA16	4.15	2.78	6.03	5.87	5.95	4.99	5.21	8.85	6.98
OfHDA17	0.43	0.37	0.54	1.27	1.32	0.18	0.82	0.34	0.42
OfHDT1	34.43	17.24	19.35	19.19	15.77	20.47	19.39	10.61	12.34
OfHDT2	50.64	11.38	7.31	32.66	10.20	19.45	28.08	7.06	6.13
OfHDT3	44.16	8.23	1.10	21.19	3.27	41.53	15.80	1.41	1.53
OfHDT5	0.49	0.00	0.74	1.89	1.19	0.00	1.98	0.83	0.75
OfHDT7	84.51	28.56	18.36	45.14	15.82	33.62	40.44	11.92	10.92
OfSRT1	2.17	1.84	2.33	1.77	1.48	1.45	2.30	2.02	2.21
OfSRT2	1.16	0.37	0.87	1.23	0.71	0.54	0.98	1.01	0.45

OfSRT3	2.52	0.51	1.34	1.77	1.11	1.06	1.64	0.90	0.59

Table S5. Gene primers used in the study.

Gene	Forward primer sequences (5'–3')	Reverse primer sequences (5'–3')
OfHAC7	CCTCCTTACCTGTTCCGTCTG	CATCTTTACACCTGTGCCATCG
OfHAG9	GAAACACCACTCCGTAGCCG	CAACATACGCAAGGAACGAAAT
OfHDMA4	GCCGTCGTAAATAATGCCTCAG	AGAGATGGCTGGGAATTCGC
OfHDT7	CAAACCAGCAGACTCCTAAATCAGC	CCACTGCTGTGCTTAGCCTTTG
OfJMJ19	GATTATGTTGCCTACAGCACTCC	GGTGATGCTGACTTGGAGGTAG
OfSDG55	TACCACTCAAGGCTGGCGAAC	TGGCAGGTGGAAGAGGTTGTC
OfSRT3	ACTTAGTGGTATTTACAGGAGCAGG	TATGAGTTACACTTGGCATCGC
OfRAN1	AGAACCGACAGGTGAAGGCAA	TGGCAAGGTACAGAAAGGGCT
